# Who Can Avoid the Heat? Inter‐Individual Differences in 24‐hr Heat Exposure Reveal Disparities in Heat Adaptation and Stress

**DOI:** 10.1029/2026GH001914

**Published:** 2026-09-12

**Authors:** G. Guzman‐Echavarria, A. Middel, N. Ravanelli, J. B. Rosales Chavez, A. R. Caldwell, C. Metzler, K. Rentz, M. Munoz, M. Guardaro, J. K. Vanos

**Affiliations:** ^1^ School of Geographical Sciences and Urban Planning Arizona State University Tempe AZ USA; ^2^ The GAME School Arizona State University Tempe AZ USA; ^3^ Heat and Health Research Centre, Faculty of Medicine and Health University of Sydney Sydney Australia; ^4^ Department of Biostatistics Fay W. Boozman College of Public Health University of Arkansas for Medical Sciences Northwest Springdale AR USA; ^5^ School of Sustainability Arizona State University Tempe AZ USA

**Keywords:** extreme heat, personal heat exposure, heat stress, heat adaptation, human heat balance, vulnerability

## Abstract

Differences in adaptive capacity and sensitivity shape behavioral and physiological responses to overheated environments, which are associated with unequal personal heat exposure (PHE). Although growing evidence documents heterogeneity in PHE across regions and populations, studies rarely examine how adaptive factors influence sub‐daily exposure patterns and resulting heat stress relative to regional heat. This study assesses intra‐ and inter‐individual variation in 24‐hr PHE and heat loads using regional weather as a non‐behavioral baseline. We studied 34 adults aged 45–74 from diverse socioeconomic, occupational, and housing backgrounds in the Phoenix Metropolitan Area. Using the HeatSuite™ system and Kestrel Drop D2 sensors, we monitored 10‐min indoor and on‐body ambient conditions over 21‐day periods during summer 2024, reconstructing PHE based on participants' locations. We used Generalized Additive Models to explain inter‐individual differences and Linear Mixed Models to examine associations with vulnerability features. Heat stress was assessed using a physiology‐based human heat balance model. Results reveal significant inter‐individual differences in individually experienced temperatures (IET) and humidities (IEH), with three sub‐daily patterns emerging: highest overall exposures, strong diurnal amplitude, and day‐long stable exposures. Median IET and IEH were 25.9°C and 1.4 kPa, with maxima reaching 47.5°C and 5.1 kPa. On average, participants experienced temperatures 10.4°C below regional conditions, with low evaporative cooling requirements to attain heat balance for sedentary activities. However, participants with income <$40k/year experienced higher peak daily temperatures. These findings demonstrate how high‐resolution personal monitoring reveals when and why heat interventions may be most effective, informing policy with locally‐grounded evidence.

## Plain Language Summary

People adapt to extreme heat through automatic body responses and protective behaviors, but their ability to stay safe depends on available resources and personal sensitivity. While people within the city experience different heat levels, we know less about how exposure changes over time, how individuals adjust to regional weather, and what this means for the body. We studied 34 middle‐aged/older adults in Phoenix, Arizona, during the hottest summer on record. Over 21 days per participant, we continuously measured personal temperature and humidity using sensors in their homes and on their bodies, with a smartwatch identifying when participants were at home. We combined these data with statistical and physical models to compare participants and estimate heat load at rest. On average, participants experienced moderate heat and reduced their personal temperature exposure by 10.4°C compared to regional conditions. During about half of participant‐hours, low evaporative cooling requirements were needed to maintain heat balance for sedentary activities. However, exposures varied widely throughout the day, and participants with lower incomes often experienced higher heat, while others experienced more stable, indoor‐controlled environments. These findings highlight the value of 24‐hr personal monitoring to better understand heat exposure and inform more effective mitigation and safety strategies.

## Key Points

- Participants with restricted financial capacity experienced higher exposures, highlighting affordability inequities in heat adaptation
- On average, participants experienced temperatures 10.4°C below regional conditions, with low evaporative cooling requirements during sedentary activities
- Sub‐diurnal exposure patterns reveal intra‐ and inter‐individual behavioral adaptation details missed by daily averages

## 1 Introduction

As overheating continues to pose a threat to health (Ebi et al., 2020; Romanello et al., 2025; Vicedo‐Cabrera et al., 2021), any effort to boost adaptation, or “the adjustment process to actual or expected heat and its effects,” remains critical (Modified from IPCC, 2022). At the individual level, this process reflects physiological (i.e., autonomic) and behavioral thermoregulation, enabling heat balance and stable internal temperatures (Romanovsky, 2018; Schlader & Vargas, 2019). Protective behaviors as voluntary adaptive responses (i.e., cool‐seeking) range from self‐adjustments, such as activity pacing or seeking shade, to actions that require greater logistics, such as access to mechanically cooled spaces. Across individuals, behavioral feasibility and effectiveness vary with the resources (social, financial, physical, and infrastructural) used to anticipate, cope with, and reduce potential harm, constituting their adaptive capacity (Guardaro et al., 2022; Hayden et al., 2011). However, heat vulnerability is also shaped by personal sensitivity, or those characteristics that increase susceptibility to heat‐related harm, such as older age and chronic health conditions (Ebi et al., 2021; Wilhelmi & Hayden, 2010). Therefore, examining how people are exposed to and cope with heat everyday provides novel insights to improve thermal well‐being in overheated environments.

Personal heat exposure (PHE), defined as direct contact between an individual and their thermal environment, lies at the center of the pathway linking heat as a hazard to adverse health outcomes (Kuras et al., 2017). While regional overheating establishes the baseline hazard, human behavior mediates how this heat is translated into personal exposure, heat stress, physiological strain, and, potentially, heat‐related illness. Given an exposure, thermal discomfort drives the voluntary decision to act and restore a preferred thermal state (Schlader & Vargas, 2019), creating a feedback loop in which behavior can either mitigate or exacerbate heat impacts (Gagnon, Schlader, & Jay, 2026). At rest, behaviors can occur even before other thermal responses (i.e., active vasodilation and sweating) (Schlader et al., 2017). However, due to motivational conflicts and interactions between physiology and perception, high exposures may arise in contexts where heat is anticipated or accepted (e.g., tourism or sports), structurally unavoidable (e.g., occupational or military settings), or individuals have impairments in thermal sensitivity (Périard et al., 2022; Rutty & Scott, 2015; Snopkowski et al., 2021; Vargas et al., 2023).

While regional weather stations, gridded meteorological products, and satellite observations provide valuable information on environmental heat, they generally represent outdoor conditions at fixed locations and cannot directly capture the environments experienced throughout daily life (Kuras et al., 2017). In contrast, PHE observations characterize heat across individual's activity spaces (Cagney et al., 2020), integrating indoor and outdoor environments, capturing fine‐scale temporal variability, and enabling direct assessment of exposure misclassification associated with ambient metrics. By linking exposures to the contexts in which they occur, PHE data also provide insights into the environmental, social, and behavioral factors shaping experienced heat. However, despite these advantages, interrelated challenges in measurement, interpretation, and representation limit their ability to inform heat adaptation (Kuras et al., 2017; Nazarian & Lee, 2021; Vecellio & Vanos, 2024). Measurement challenges arise from heterogeneity in measured variables, sensor types, body placement, and sampling strategies. Simultaneously, the increasing use of wearable sensors has improved spatial representativeness and made these uncertainties explicit (Bailey et al., 2020; Habeeb et al., 2022). Interpretation challenges to relate observed exposures to their causes and consequences further arise from choices of heat exposure metrics (i.e., intensity, frequency, or duration), statistical approaches, and contextual data (i.e., surveys, interviews, physiological markers). Lastly, the generalizability of PHE findings remains limited within and across contexts. Within specific locations, the high logistical and resource demands of PHE assessments constrain population‐representative sampling (Hondula et al., 2021; Milando et al., 2022). On a global scale, existing studies are disproportionately concentrated in the global north (Nazarian & Lee, 2021). Recently, a growing number of studies have spanned wider geographies and populations (e.g., Butler‐Dawson et al., 2024; Deshpande et al., 2024), including larger sample sizes (e.g., Meng et al., 2025; Xia et al., 2024), and have begun linking exposures to stress and strain markers (e.g., Lin et al., 2022; Milando et al., 2022; Sugg et al., 2022).

Overall, research consistently demonstrates substantial heterogeneity in PHE within case studies, confirming that differential adaptive capacity and vulnerability translate into unequal exposure profiles. Using qualitative and quantitative approaches, studies account for adaptive capacity factors that are either generic (i.e., related to broader social, political, and economic stressors, embedding structural constraints) or heat‐specific (e.g., constraints on indoor and outdoor exposure, air conditioning (AC) access and use) (Guardaro et al., 2022). Baseline and post‐monitoring surveys and interviews collect participant‐level information, while time‐activity daily logs, thermal comfort ratings, and GPS tracking enable concurrent assessment of activity patterns and adaptive responses (i.e., behaviors) in relation to heat exposure. Higher PHE is generally observed among individuals with fewer resources to cope, including housing, limited or ineffective AC use, and also among those with greater engagement in outdoor or indoor heat‐generating activities (Deshpande et al., 2024; Han et al., 2025; Hondula et al., 2021; E. Kuras et al., 2015; Milà et al., 2020; Milando et al., 2022). In occupational studies, peak exposures are reported among those performing prolonged outdoor or heat‐intensive work with limited opportunities for behavioral modification (Butler‐Dawson et al., 2024; Sugg et al., 2022; Uejio et al., 2018). Conversely, lower PHE is more common among individuals who can access and spend more time in cooler environments, or adjust daily activities to avoid peak heat (Hass & Ellis, 2019; Hondula et al., 2021; Milando et al., 2022). However, even in AC‐rich contexts, exposure may remain elevated due to constrained or selective use (Wright et al., 2020). In settings with low AC prevalence, housing characteristics may play a more substantial role in shaping exposure (Weitz et al., 2022).

In PHE research, regional ambient temperature data have been used in three main ways: as a background reference, to quantify exposure misclassification when ambient conditions are assumed to represent individual exposures, and, more recently, as predictors of PHE itself (Xia et al., 2024). Across geographical contexts, studies consistently report a disconnect between personal temperature and ambient conditions measured by weather stations or gridded data sets (Hass et al., 2022; Meng et al., 2025). Ambient temperatures may underestimate PHE when heat is retained or generated indoors, particularly at night (Han et al., 2025; Milà et al., 2020), or when cold weather prevails (Meng et al., 2025). Conversely, ambient temperatures tend to overestimate PHE when individuals can avoid outdoor heat through behavioral or structural cooling (Bernhard et al., 2015). This latter effect is especially true for very hot‐arid cities, where large diurnal temperature ranges and indoor‐outdoor contrasts shape daily exposure patterns, imposing sustained heat‐avoidance behaviors during summer (Hondula et al., 2021; Wright et al., 2020). Adaptive capacity to heat and broader heat vulnerability further shape these deviations. In Phoenix, for example, ambient temperature often overestimates exposure for middle‐income residents who use AC and underestimates indoor exposure for low‐income residents with constrained AC use (Zhao et al., 2021). In summary, PHE data contain information reflecting the behaviors or reactions individuals make to modify (i.e., reactive approach) or avoid (i.e., preventive approach) high heat loads, thereby shaping their exposures. Therefore, beyond estimating misclassification errors, regional heat can serve as a reference signal for comparing individuals living in the same city (i.e., personal deviations from regional heat). This approach provides an opportunity to study how individuals modify their exposures in relation to broad vulnerability features or specific behaviors, depending on data availability.

*This study builds on the premise that PHE during hot weather and corresponding deviations from regional heat levels can be interpreted as partial evidence of behavioral adaptation (via environmental selection and modification) within individual activity spaces*. *Specifically*, *we examine how sub‐diurnal PHE data differ among individuals experiencing similar regional weather*. Overall, we seek to demonstrate how PHE emerges from the interaction between regional heat and broad heat vulnerability (i.e., adaptive capacity and sensitivity) and propose an approach to interpret differential heat stress (i.e., personal heat loads) among individuals. We address the following research question:

How does the sub‐diurnal variability of PHE and heat stress differ across participants as a function of baseline regional weather, time of day, and heat vulnerability?

Specifically, we aim to: (a) Reconstruct hourly PHE in terms of Individually Experienced Temperature (IET) and Humidity (IEH) to describe sub‐diurnal variability across participants; (b) Quantify sub‐diurnal deviations of PHE from regional weather (ΔIET, ΔIEH); (c) assess whether, and how, inter‐individual differences on IET relate to regional heat and time of day; (d) evaluate the influence of vulnerability, sensitivity, and adaptive capacity features on daily PHE metrics; and (e) compare heat stress estimates from regional weather and interpersonal PHE with reference to metabolic heat from rest and sedentary‐light activities.

## 2 Materials and Methods

**Figure 1:**
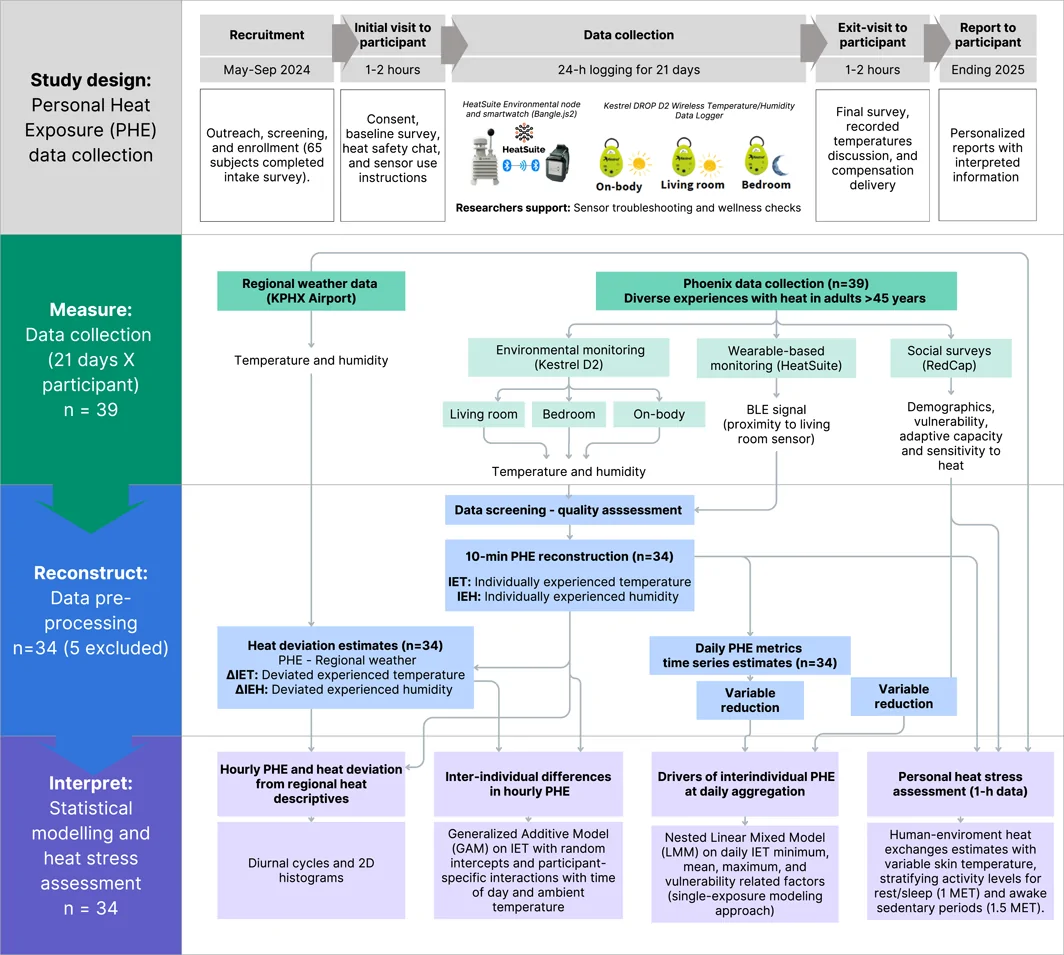
Methodological workflow of the mixed‐methods study design, showing the research protocol from a participant perspective (upper gray panel), and data pipeline and participants retained from collection (Section 2.2, 2.3 and Text S1, S2 in Supporting Information S1), continuing with pre‐processing (Section 2.3 and Texts S3–S5 in Supporting Information S1) to formal analysis (Section 2.4, 2.5 and Text S6 in Supporting Information S1). Arrows indicate input and output data from each sub‐step, with text bolded for processes. Formal analysis in this study involves descriptive statistics, statistical modeling to account for hierarchical data structure and non‐simultaneous records, and a heat stress assessment. Human‐environmental heat exchanges were estimated using a modified implementation of the ASU/USYD HEAT‐Lim Human‐Environmental Adaptation and Threshold Limits model (Guzman‐Echavarria & Vanos, 2025).

This study is part of the NSF Global Center Heat Adaptation (GCHA) studies network in the United States, Canada, Australia, and Colombia, using HeatSuite technology to advance methods for assessing personal‐ and household‐level heat exposure, risk, and adaptation. HeatSuite is a low‐cost, fully data‐governed multimodal sensor platform that monitors environmental conditions and physiological and behavioral responses in free‐living individuals (Ravanelli et al., 2025). In this PHE‐focused study (Figure 1), we restrict the analyses to environmental exposure data and exclude physiological and ecological momentary assessment (EMA), which are reserved for complementary research. The research protocol was approved by the Arizona State University Institutional Review Board (STUDY00019657).

### 2.1 Study Location and Regional Weather

This study was conducted from 4 June to 17 October 2024, in the greater Phoenix Metropolitan Area (Arizona, USA), a hot‐arid region (Köppen–Geiger *BWh*) where summertime temperatures frequently exceed 40°C and is home to approximately 5 million residents (MAG & Maricopa Association of Governments, 2025). Despite high residential AC prevalence (∼95%), indoor heat exposure remains a significant concern, as AC availability does not consistently ensure safe indoor conditions, particularly in older housing stock or during system malfunction (Baniassadi et al., 2019; Fraser et al., 2017). Recent heat‐related mortality records show that ∼25% of heat‐related deaths occur indoors in uncooled environments (95% individuals older than 50 years) (MCDPH, 2024, 2025), with exacerbated risk toward manufactured housing (recreational vehicles (RVs), trailers, mobile homes) that represents a small fraction of the housing stock (∼5%) (Charley et al., 2023).

**Figure 2:**
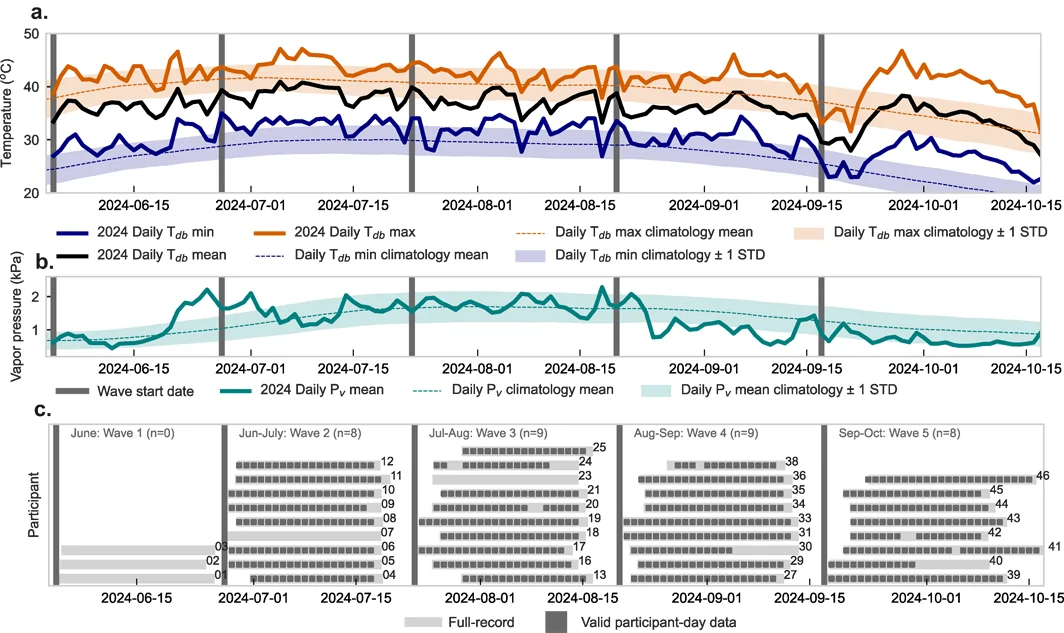
Daily temperature and absolute humidity at Phoenix Sky Harbor Airport (KPHX) and corresponding participant data collection timelines (June–October 2024). (a) Daily minimum, mean, and maximum dry bulb temperatures (*T*_db_, °C). (b) Daily mean vapor pressure (*P*_*v*_, kPa). For (a) and (b), dotted lines and shaded bands represent the 2005–2020 daily climatology (mean ± standard deviation (STD)), smoothed using a 30‐day centered rolling window. (c) Gantt‐style timelines of participant data inclusion after the screening process. Gray vertical lines show the start of each measurement wave, labeled with the number of participants retained after data screening.

The summer of 2024 was the hottest on record in the Phoenix region (Figure 2), with persistent hot and dry conditions extending into October following a delayed North American Monsoon (NWS Phoenix, 2025). Observations from the Phoenix Sky Harbor International Airport (KPHX) weather station reported a mean dry‐bulb temperature (*T*_db_) of 36.2 ± 4.9°C (range: 21.7–48.0°C), with average daily minimum and maximum *T*_db_ values of 30.1 ± 3.0°C and 41.9 ± 2.7°C, respectively. Mean water vapor pressure (*P*_*v*_) was 1.22 ± 0.55 kPa, corresponding to an average relative humidity of 21.2 ± 11.4%.

### 2.2 Study Design and Sample Characteristics

We employed a purposive, convenience‐based sampling approach to recruit middle‐aged and older adults with diverse lived experiences, without aiming for population‐representative estimates, given the expected sample size. Eligibility criteria included age ≥45 years; residence in single‐unit, multi‐unit, or manufactured housing; presence in the local area during summer; and ability to read and understand English or Spanish. During the 21‐day data collection period, we visited participants' homes at least twice. On the first visit, we obtained written informed consent, administered a baseline survey, installed the sensors, and trained participants on their use. On the final day, we administered a second survey and provided economic compensation. Additional details on recruitment and study procedures are provided in Text S1 and S2 in Supporting Information S1.

Initial enrollment included 65 individuals; 39 provided informed consent and completed the study, and 34 participants were retained after data screening (Table 1). Participants' ages ranged from 45 to 74 years old, with a female‐to‐male ratio of 7:3, 36% Spanish‐speakers, household income ranged from $10,001‐$20,000 to over $200,000, with a median of $60,000 to $80,000; most participants were homeowners, and all had some cooling equipment at home (some multiple types). The proportions of housing types in our sample are similar to those reported for sites of indoor‐heat‐related deaths (MCDPH, 2025). Compared to the U.S. Census American Community Survey 2019–2023 in the Maricopa Association of Governments (MAG) area, our sample matches the proportions of ethnic representation and homeownership but overrepresents low‐income households and manufactured home dwellers.

**Table 1:** Participant Demographics, Heat Coping Narrative, and Specific Adaptive Factors to Heat Evaluated in This Study (*N* = 34)

	N	(%)
Age: M ± SD	59.7	±7.90
Sex:		
Female	23	(67.6)
Male	10	(29.4)
NA (missing record‐no answer)	1	(2.9)
Race/Ethnicity		
Non‐Hispanic White	21	(69.7)
Hispanic/Latino/Mexican‐American	11	(32.3)
Native American	2	(5.8)
Income^a^		
Less than 40 K/year	12	(35.3)
40–80 K/year	11	(32.4)
More than 80 K/year	10	(29.4)
NA	1	(2.9)
AZ Social Vulnerability Index (AZ SVI)	0.539	±0.24
Heat coping narrative^b^		
Heat is an inconvenience	26	(76.5)
Heat is a manageable problem	5	(14.7)
Heat is a disaster or a catastrophe	3	(8.8)
Housing type		
Single‐family home detached/attached	22	(64.7)
Apartment in a 2‐4 story building	4	(11.8)
Manufactured home	8	(23.5)
Home ownership		
Yes	24	(70.6)
No (include paying rent, and other occupation type)	10	(29.4)
Central air conditioning (AC)^c^		
Yes	27	(79.4)
No (including wall/window AC, mini‐splits, and evaporative (swamp) cooler)	7	(20.6)
Limitations on AC use during data collection		
Yes	30	(88.2)
No	3	(8.8)
NA	1	(2.9)
Limitations to afford AC		
Yes	9	(25.5)
No	25	(73.5)
Fan use		
Yes	28	(82.4)
No	6	(17.6)
Main daily activity at outdoors		
Yes	23	(67.6)
No	10	(29.4)
NA	1	(2.9)
Heat‐risk comorbidity^d^		
Yes	19	(55.9)
No	15	(44.1)

^a^ Income recoded to obtain equally sized groups.

^b^ Guardaro et al. (2022).

^c^ Primary cooling method at home.

^d^ Heat‐risk comorbidity if any of the following groups of illnesses were reported: hypertension, diabetes, stroke/CVA, cardiovascular, respiratory, or kidney disease.

### 2.3 Data Collection and Pre‐Processing

#### 2.3.1 Regional Heat and Heat Deviation

We assumed outdoor regional heat as a reference for interindividual comparisons, serving as a proxy for counterfactual exposures in the absence of behavioral environmental selection. In doing so, a common regional hazard is assumed for all participants; thus, translating intra‐city thermal variability into a personal exposure signal. This is valid because thermal variability at the local scale (i.e., neighborhoods) depends on urban structures and cover types (Stewart & Oke, 2012), and higher outdoor temperatures often occur in neighborhoods with higher impervious surfaces, a lack of shade, and lower socioeconomic status (Harlan et al., 2006). Therefore, intra‐urban heat variations themselves contain information about individuals' adaptive capacity to heat. To operationalize that baseline exposure, we used fixed‐point *T*_db_ and *P*_*v*_ observations from a fully open space (KPHX standard weather station; Figure 2). Finally, we estimate heat deviation from regional heat as the difference between PHE minus ambient records for both *T*_db_ (ΔIET) and *P*_*v*_ (ΔIEH). Given sustained high regional temperatures, we assume that negative values are primarily associated with cool‐seeking behaviors aimed at avoiding or mitigating heat stress.

#### 2.3.2 Personal Heat Exposure

We conducted measurements across five waves (time periods) from June to October due to limited sensor availability (10 HeatSuite kits; Figure 2c). Across waves, daily regional *T*_db_ varied from 33.5 to 38.8°C, and *P*_*v*_ from 0.7 to 1.75 kPa (14.6%–29.1%). Each participant was monitored for 21 days using a HeatSuite kit and three Kestrel DROP D2 wireless temperature‐humidity loggers, recording *T*_db_ (IET) and humidity converted to vapor pressure *P*_*v*_ (IEH) at a 10‐min logging interval. Relative humidity was not used in the analysis due to its temperature dependence (Baldwin et al., 2024). To ensure consistency across sensor types, only Kestrel data were used, since they captured indoor and outdoor exposures. Kestrel D2 sensors have an accuracy of ±0.5°C and ±2% for temperature and relative humidity, respectively, and exhibit the smallest bias among commercial sensors commonly used in PHE studies (Bailey et al., 2020; Habeeb et al., 2022).

One Kestrel sensor was placed in the primary living space adjacent to the HeatSuite environmental node; a second was placed in the bedroom; and a third was carried by participants (attached to a bag or belt loop) to capture outdoor exposure. Participants were instructed to maintain usual activities while wearing the on‐body Kestrel and HeatSuite smartwatch (Bangle.js 2). Bluetooth Low Energy (BLE) proximity between the smartwatch and the Heatsuite node was used to detect when participants were at home. This protocol produced three IET and IEH time series (living room, bedroom, on‐body), which were integrated into a reconstructed PHE profile using self‐reported wake and sleep times and BLE proximity data (See Text S3 in Supporting Information S1 for PHE reconstruction details).

Data screening followed procedures similar to Hondula et al. (2021). Values were winsorized at the 0.1st or 99.9th percentile by sensor and variable, and participant‐days with >3 hr of missing data were excluded to reduce bias in daily PHE metrics. To address potential solar‐heating bias of on‐body Kestrel Drop D2 measurements in the absence of personal radiation observations, we compared IET against KPHX records using the mean temperature bias (+2.74°C) reported by Habeeb et al. (2022) for backpack‐mounted Kestrel sensors (Figure 7a). This threshold was used to identify observations potentially affected by sensor heating. On‐body IET values exceeding regional *T*_db_ by more than 2.74°C were flagged and capped at *T*_db_ + 2.74°C between sunrise (solar elevation >15°) and 1 hr after sunset, affecting 430 observations (0.4% of all records). Reported bias on belt loop on‐body placement is minor, but we stick to backpack information to be conservative. After screening, 34 of 39 participants were retained, providing 97.8% valid records (Figure 2c). No participants from wave 1 (*n* = 3) were retained due to complete data loss, and two participants were excluded due to unrealistic variability in their IET data.

To evaluate different dimensions of PHE (magnitude, frequency, duration, and variability) in relation to adaptive capacity to heat, we compute daily metrics from 10‐min data, following Hondula et al. (2021), adding diurnal range and daytime/nighttime variance (Table S1 in Supporting Information S1). After redundancy and linearity analyses, we retained three metrics: daily IET_min_, IET_mean_, and IET_max_ (See Text S4 in Supporting Information S1 for details on redundancy analysis and inter‐individual variability). Pre‐processing procedures (Figure 1) were implemented in Python v3.9.7.

#### 2.3.3 Adaptive Capacity, Sensitivity to Heat, and Broader Vulnerability

Information from adaptive capacity and sensitivity was collected from entry and exit surveys administered in REDCap (Harris et al., 2019), adapted from prior Phoenix‐based instruments (Hayden et al., 2011; Wright et al., 2020). Surveys captured baseline resources for coping with summertime heat and predictors of indoor exposure, including familiarity with the city, housing and built environment characteristics, expected and preferred heat exposure, prior heat‐health outcomes, social capital, and demographics. Surveys were available in English and Spanish. Broader social vulnerability was assigned to each individual based on the Arizona Social Vulnerability Index (AZSVI) score associated with their residence address (Arizona Department of Health Services, 2024).

From the full survey instrument, we selected demographic and adaptive factors previously associated with IET and indoor temperatures in Phoenix (Hondula et al., 2021; Larsen et al., 2022; Wright et al., 2020), and additionally included age, heat‐coping narrative (Guardaro et al., 2022), and heat‐risk comorbidities. To identify factors providing unique information in this sample, we conducted a redundant analysis: we removed variables that were predictable from others (e.g., house size from income) using the R package *rms* and an *R* ^2^ threshold of 0.5, and used Hoeffding's D clustering for confirmation. As a result, we retained 12 factors (10 categorical, 2 continuous) (See Table 1). See Text S5 in Supporting Information S1 for the full factor list and details of the redundancy analysis.

### 2.4 Statistical Analysis

First, we describe magnitudes, ranges, and time‐of‐day patterns of PHE and their deviations from regional weather conditions. Descriptive analyses include diurnal cycles per participant to display sub‐diurnal variability and univariate and bivariate histograms to examine paired humidity and temperature within both PHE and regional weather. Daytime/nighttime were separated based on sun elevation (>0°). Later, we used statistical models to (a) enable inter‐individual comparisons of personal heat exposure under different levels of regional heat and (b) characterize associations between personal heat exposure metrics and indicators of adaptive capacity, sensitivity, and vulnerability.

We evaluated sub‐daily (10‐min and 1‐H) and daily PHE data model performance using linear mixed‐effects (LMM) and generalized additive models (GAMs) with Gaussian error structure, including participant‐level random effects to account for the hierarchical structure of the cohorts. We found that linear models were not suitable for 10‐min and 1‐H data due to heteroscedasticity and heavy‐tailed residuals, but they were suitable for daily aggregate data. We retain both the GAM and LMM approaches. First, a GAM applied on IET to describe inter‐individual differences in hourly PHE (Section 3.3), retaining a Gaussian distribution, despite a scaled‐t yielding a slightly better fit to ensure consistency with variance‐based inference (e.g., intraclass correlation coefficient estimation (ICC)). Over LMM, GAMs capture non‐linear associations and cyclic time‐of‐day patterns in IET. Second, we used a single‐exposure modeling approach, fitting separate Nested LMM models for each combination of daily PHE metric (three metrics) and vulnerability‐related factors (12 factors). This approach allowed examination of individual associations without simultaneous adjustment for correlated factors. *P*‐values from Type II omnibus Wald χ^2^ tests of all single‐exposure models (36 in total) were adjusted using the Benjamini–Hochberg false discovery rate (FDR) procedure. Factors with *q* < 0.05 were considered significant after FDR correction, whereas factors with 0.05 ≤ *q* < 0.10 were considered suggestive. For specific details on PHE metrics and factors retained in the analysis, see Text S4 and S5 in Supporting Information S1; model implementation and test results are presented in Text S6 and Table S3 in Supporting Information S1. Modeling was conducted in R using the *lmerTest* (Kuznetsova et al., 2017) and *mgcv* (Wood, 2011) packages.

### 2.5 Heat Stress Assessment

We assessed potential heat stress under regional weather conditions (non‐behavioral change scenario) and observed PHE (behavioral change scenario), assuming seeking cooling as the predominant behavioral change. This assessment evaluated whether combinations of IET and IEH permitted dissipation of metabolic heat during sedentary activity primarily via sensible heat transfer, without requiring evaporative cooling via sweating. Specifically, we evaluated evaporative requirements for heat balance (*E*_req_; largely driven by *T*_db_ in the absence of additional radiative load) relative to evaporative heat loss capacity ($E_{\max_{\lim}}$; environmentally driven by *P*_*v*_) as in Guzman‐Echavarria et al. (2022).

Human‐environmental heat exchanges for steady‐state 1‐hr exposures were estimated using the Python implementation of the ASU/USYD Human‐Environmental Adaptation and Threshold Limits (HEAT‐Lim) model (Guzman‐Echavarria & Vanos, 2025; Vanos et al., 2023). In this application, we relax the full vasodilation assumptions used in HEAT‐Lim, Guzman‐Echavarria et al. (2022) since most exposures were low‐to‐moderate in the observed PHE records. Instead of a fixed maximum skin temperature value, we adopted a variable skin temperature for steady‐state exposures from Mehnert et al. (2000) assuming a core temperature of 37°C. Estimates were stratified by self‐reported awake and sleep periods (see Text S3 in Supporting Information S1 for time stratification details), with metabolic rates of 1.5 MET (sedentary‐light activity) and 1.0 MET (at rest), respectively.

Physiological and environmental parameters were conservatively specified: actual participant's body mass (80.9 ± 20.4 kg) and Dubois surface area (1.87 ± 0.20 m^2^), maximum skin wettedness and sweat rate averaged between “young” and “older” adult profiles in Vanos et al. (2023) ($\omega_{\max}$: 0.75, ${\text{SR}}_{\max}$: 0.63 L/hr), mean radiant temperature equal to IET or regional *T*_db_, and wind speed 0.3 m/s. Clothing insulation and effective radiative area (*A*_eff_) were defined by activity state: awake (0.36 clo; *A*_eff_ = 0.73) standing in light shorts and a cotton T‐shirt (Smallcombe et al., 2021), and sleep (0.6 clo; *A*_eff_ = 0.61) as supine on bed, short‐sleeved t‐shirt, pants, and blanket (Lan et al., 2018).

## 3 Results

Each subsection presents results from 1‐hr aggregated data, describing median (interquartile range), unless otherwise noted.

### 3.1 Sub‐Diurnal Variability in PHE Across Participants

**Figure 3:**
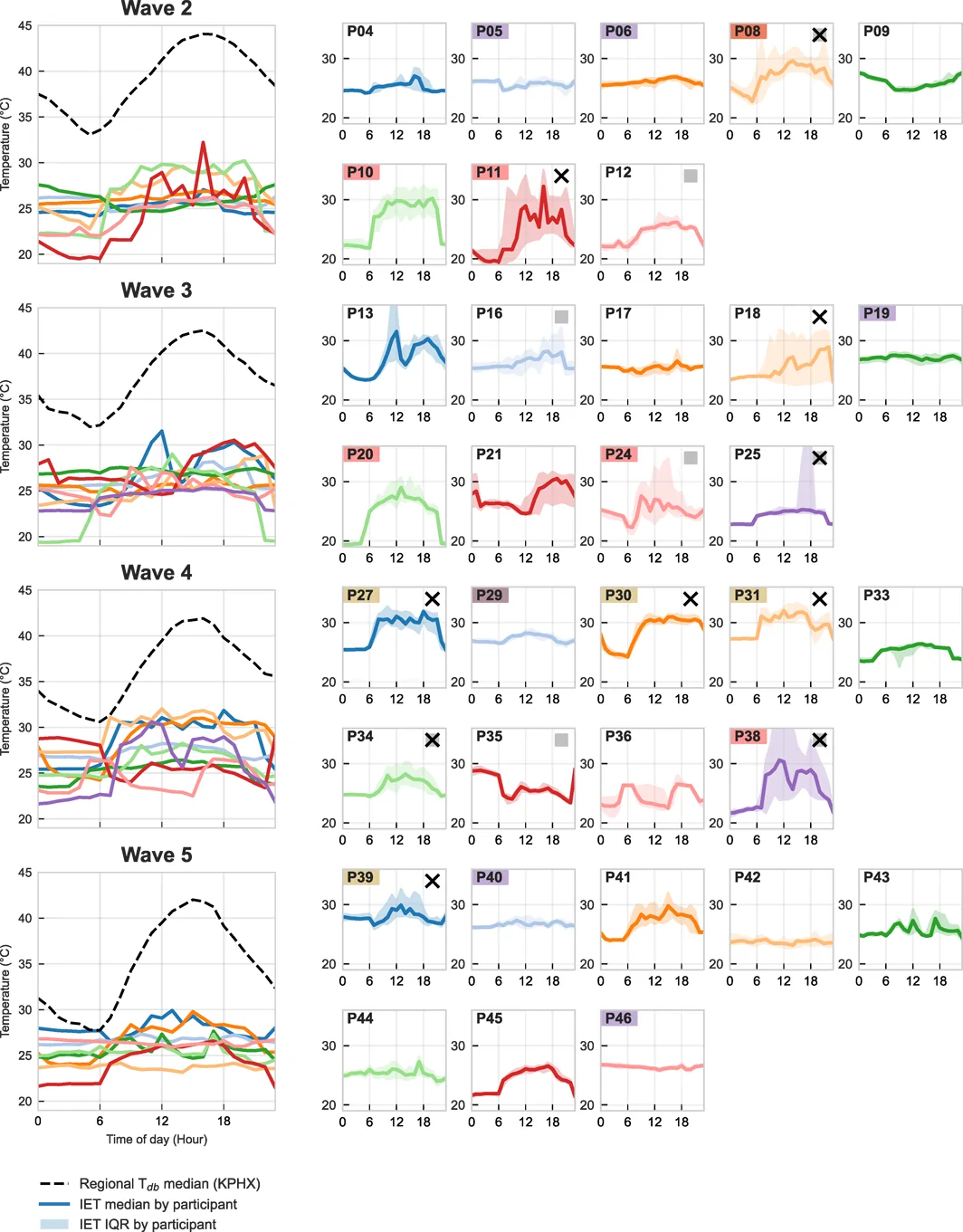
Participant‐level diurnal cycles observed 1‐hr IET by measurement wave (left), with regional dry bulb temperature (*T*_db_) (°C) shown as dashed black lines. Right: Individual‐level IET diurnal cycles with markers for participants living in areas with high AZSVI scores (gray squares), self‐reported income <$40k/year (black crosses), and participants selected by having the *highest overall exposure* (gold), *strongest diurnal amplitude* (red), or *stable day‐long exposure* (violet). Solid lines and shaded bands represent the hourly media IQR. After data screening, no participants were retained from wave 1.

Inter‐individual variations in IET (Figure 3) and IEH (Figure S4 in Supporting Information S1) were substantial, with larger daytime‐nighttime contrasts in IET than in IEH (Table 2). Across participants, median IET ranged from 22.9°C to 29.9°C, compared with an all‐data median of 25.9°C (24.5–27.5°C) (Figure 6a). At the sample level, daytime IET was on average 0.9°C higher than nighttime and 1.3°C higher than sleeping hours (midnight–05:00). Participant median IET values ranged from 23.6 to 30.9°C during daytime and 19.5–28.7°C during sleeping hours. Maximum 1‐hr experienced temperatures varied widely, from 30.5°C to 47.5°C (10 participants >40°C).

**Table 2:** IET and IEH Descriptives From All‐Data Set (1 hr Aggregates) and Across Participants (Minimum, Percentiles 25, 50, 75, and Maximum Values)

	All 1H data		Participant level (*n* = 34)				
	Median	(IQR)	Minimum	P: 25th	P: 50th	P: 75th	Maximum
IET (°C)							
All 24‐hr	25.9	(24.5–27.5)	22.9	24.7	25.7	26.5	29.9
Daytime	26.3	(25.0–28.4)	23.6	25.4	26.2	27.4	30.9
Nighttime	25.4	(23.9–26.8)	19.7	23.9	25.1	26.5	28.5
Sleeping‐hours	25	(23.5–26.4)	19.5	23.8	24.9	26.2	28.7
IEH (kPa)							
All 24‐hr	1.4	(1.3–1.5)	1.2	1.3	1.4	1.5	1.8
Daytime	1.4	(1.3–1.6)	1.2	1.3	1.4	1.5	1.9
Nighttime	1.3	(1.2–1.4)	1.1	1.2	1.4	1.4	1.5
Sleeping‐hours	1.3	(1.2–1.4)	1.0	1.2	1.3	1.4	1.5

*Note*. Daytime/nighttime are depicted based on sun elevation; sleeping hours are considered from midnight to 5:00 a.m.

Median IEH varied from 1.2 to 1.8 kPa across participants, with an all‐data median of 1.4 kPa (1.3–1.5 kPa), corresponding to a median relative humidity of 41.2% (37.8%–45.0%). The most humid environments experienced ranged from 1.6 to 5.1 kPa.

Because participants spent most of their time in thermally controlled environments, daily IET and IEH minima and maxima did not consistently align with regional T_db_ or Pv. While daily maximum IET occurred in the afternoon for many participants, others reached their highest IET around midnight (e.g., P35, P46, P06, P09), and daily minimum occurred at varying times. As a result, the daily minimum and maximum IET ranges nearly overlap. Diurnal amplitudes were also highly variable, with median ranges of 7.2°C (4.7–9.3°C) for IET and 0.51 kPa (0.4–0.9 kPa) for IEH.

### 3.2 Distinct Diurnal Exposure PHE Profiles

**Figure 4:**
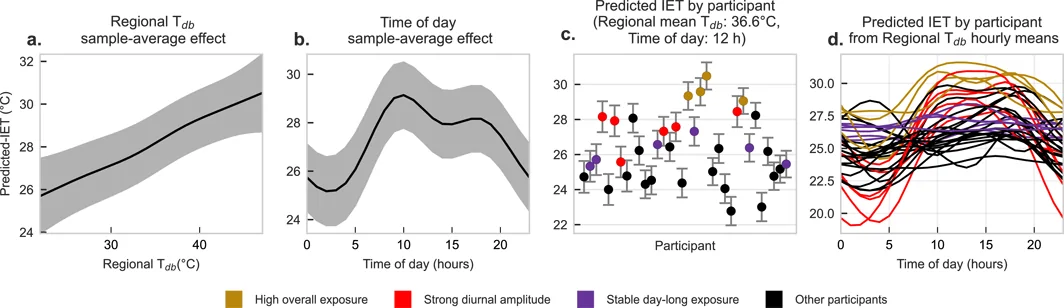
Decomposition of IET by fixed and random effects by participant from the GAM model applied to 1‐hr aggregates. Panels (a) and (b) show sample‐average effects of regional temperature and time of day (Solid line: estimates, Bands: 95% Confidence interval CI). Panel (c) shows individual‐level adjusted predictions (CI = 95%) centered at the data set mean regional *T*_db_ (36.6°C) and time of day (12 hr). Panel (d) presents adjusted 24‐hr IET predictions using regional *T*_db_ hourly averages. (c) and (d) illustrate inter‐individual differences as if participants were exposed to the same regional weather. Colors in (c) and (d) indicate selected participants by having the *highest overall exposure* (gold), *the strongest diurnal amplitude* (red), or *stable day‐long exposures* (violet); participants not included in any of the groups are shown in black.

Despite heterogeneity in exposures, three recurring exposure patterns emerged based on within‐day IET magnitude and timing. To illustrate these patterns, we selected the six participants with the highest median IET (“*Highest overall exposures*,” gold lines in Figures 3, 4, and 7, Figures S4, S7 in Supporting Information S1), with the largest diurnal IET amplitudes (“*Strongest diurnal amplitudes*,” red lines), and with the smallest diurnal IET amplitudes (“*Stable day‐long exposures*,” violet lines). These profiles are intended as descriptive examples of contrasting exposure patterns rather than a formal clustering process to generalize vulnerability features.

Participants with the *highest overall exposures* experienced IET between 27 and 32°C for ∼18 hr/day. They predominantly earn low incomes (5 of 6 earning <$40,000/year), live in single‐unit or manufactured housing, and reported variable thermostat settings and thermal preferences. This group included the only participant who relied on evaporative cooling as the primary cooling method, and two of the three used window air‐conditioning units.

Participants with *strong diurnal amplitude* (up to 14.9°C) exhibited frequent shifts in IET time series associated with indoor‐outdoor transitions, lived in varied housing types, used diverse cooling systems, and reported heterogeneous income levels, educational attainment, and thermal preferences. Several experienced among the lowest nighttime IETs (∼19°C, e.g., P20, P11), while their mean daily maximum IETs ranged from 34.3 to 35.9°C. Although only two reported doing their main activity outdoors, several described frequent outdoor exposures through daily activities such as gardening, public transportation use, or as part of their jobs (e.g., cleaning services).

Participants with *stable day‐long exposure* relied primarily on central AC to maintain consistent temperatures, with diurnal ranges of 2.6–4°C. Comparisons between wearable and indoor sensors indicated that these participants spent most of their time indoors at home, with only brief or infrequent departures. Most lived in single‐unit housing (except for one participant in manufactured housing), and only one participant reported limiting air‐conditioning use due to cost. Income, education level, and thermal preferences varied within this subgroup.

### 3.3 Effects of Regional Weather and Time of Day on IET

The GAM explained 50.4% of the deviance in IET. All smooth terms were significant. The population‐level smooth for regional *T*_db_ had an estimated degrees of freedom (edf) of 1.74 (*F* = 4.20, *p* = 0.015), indicating an approximately linear association between regional temperature and IET. In contrast, the population‐level smooth for time of day was more complex (edf = 3.60, *F* = 8.92, *p* < 0.001), confirming that exposure varies at 24‐hr patterns. Participant‐specific deviations from these population smooths were substantial for both regional *T*_db_ (edf = 80.5, *F* = 1.65, *p* < 0.001) and time of day (edf = 133.5, *F* = 15.8, *p* < 0.001), indicating that individuals varied meaningfully in both their thermal response to specific regional weather and their daily IET routines. Random intercepts for participants were also significant (edf = 10.7, *p* < 0.001). The ICC based on random intercepts alone was 0.12, indicating that approximately 12% of the residual variance in IET was attributable to stable between‐participant differences. When participant‐specific smooth interactions with regional *T*_db_ and time of day were included, the ICC increased to 0.34, suggesting that roughly a third of the total variance reflects individual‐level variability in thermal exposure patterns.

Based on sample‐averaged predictions, IET increased by 0.25°C (95% CI: 0.15–0.34°C) per 1°C increase in regional *T*_db_ (Figure 4a). The population‐average diurnal cycle was bimodal (Figure 4b), with a primary peak from 09:00 to 11:00 (∼29.1°C), a secondary peak from 16:00 to 18:00 (∼28°C), and a daily minimum from 01:00 to 04:00 (∼25.5°C). Adjusted predictions at mean (Regional *T*_db_ = 36.6°C and Time of day = 12hr) revealed inter‐individual variation in baseline IET ranging from 22.8°C to 30.5°C (Figure 4c). They predicted IET diurnal cycles (Figure 4d) based on hourly averages from the Regional *T*_db, which_ were consistent with observed IET from the emerging distinct profiles (Figure 7d).

### 3.4 Associations of Daily IET With Vulnerability, Sensitivity, and Adaptive Capacity Factors

The mean daily IET_min_, IET_mean_, and IET_max_ were 23.6°C, 26.9°C, and 30.8°C, respectively, based on all participant‐days at the original 10‐min temporal resolution. These metrics exhibited substantial interindividual variability (ICC = 0.7, 0.6, 0.4, respectively). We fitted 36 mixed‐effects models to characterize associations between these three PHE metrics and 10 categorical and two continuous vulnerability factors (see Section 2.3.3).

**Figure 5:**
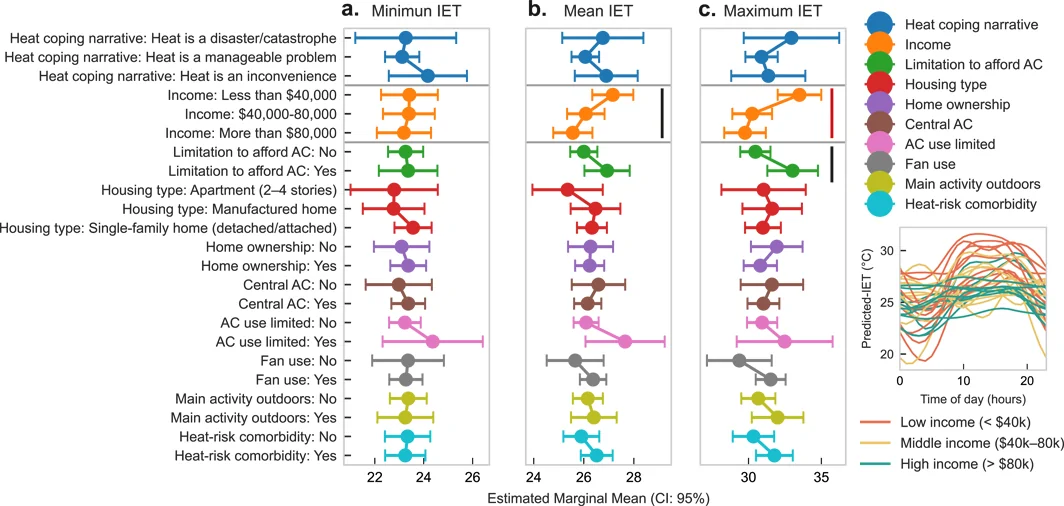
Daily minimum (a), mean (b), and maximum (c) IET group estimated marginal means by vulnerability‐related categorical factors, and participants' 24‐hr GAM adjusted IET stratified by income (d). Marginal effects in (a–c) are centered at the daily mean regional temperature, whereas the regional *T*_db_ hourly averages were input from 24‐hr IET predictions in (d). Red labels indicate adaptive factors whose omnibus tests remained significant after Benjamini–Hochberg false‐discovery‐rate correction (*q* < 0.05). Black labels indicate factors showing evidence of association before correction (*p* < 0.05) but not after FDR adjustment. Separate models were fitted for each combination of PHE metric and factor (single‐exposure model approach). Continuous factors (i.e., age and AZSVI score) are not shown in this figure.

Among the factors examined (Figure 5, Table S3 in Supporting Information S1), income was associated with IET_max_ (*P* < 0.005), after Benjamini–Hochberg false‐discovery‐rate correction (*q* = 0.012). At the mean daily regional *T*_db_ of 36.6°C, participants in the lowest income category (<$40,000) experienced 3.7°C higher IET_max_ than those earning more than $80,000 annually (95% CI: 1.25–6.23°C), and 3.2°C than those earning between $40,000 and 80,000 (95% CI: 0.81–5.68°C). Social vulnerability (AZSVI) also showed evidence of a positive association with IET_max_, with an estimated 5.1°C difference (95% CI: 1.4–8.7°C) between participants with the lowest and highest AZSVI scores. However, this association remains suggestive after FDR correction (*q* = 0.081). Before adjustments, we also found that participants reporting affordability trade‐offs to run their air conditioning experienced a 2.6°C higher IET_max_ (95% CI: 0.5–4.6°C), although this factor also did not remain significant after FDR correction (*q* = 0.120). A similar case happened to the association between daily IET_mean_ and income (*q* = 0.124).

### 3.5 PHE Under Non‐Behavioral and Behavioral Scenarios

**Figure 6:**
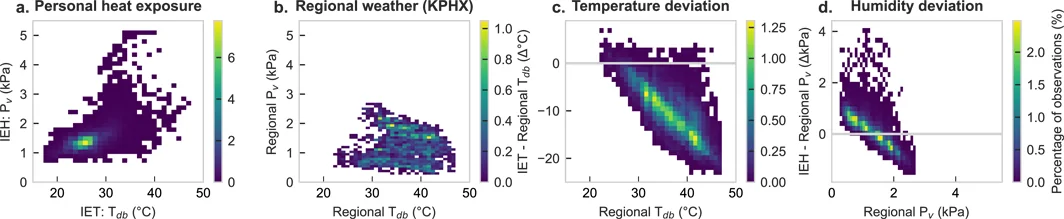
2D histograms (30 bins) showing the relative frequency of observations from 1‐hr (a) personal heat exposure (IET/IEH); (b) regional weather during the study (both plotted with *T*_db_ on the *x*‐axis and *P*_*v*_ on the *y*‐axis); and (c–d) temperature (ΔIET) and humidity (ΔIEH) deviations between personal exposure and regional weather. Panels (c) and (d) use Bland‐Altman style plots referenced to regional weather; positive/negative values indicate increases/decreases in PHE relative to the regional weather station.

Compared with a non‐action scenario (defined by exposures to regional baseline weather; Figure 6b), participants substantially reduced their heat exposure (Figure 6c). On average, participants reduced their IET substantially by 10.4°C below regional *T*_db_ (IQR: 14.0–6.8°C). IET reductions were larger during daytime hours (11.7°C) than during sleep periods (7.7°C). The magnitude of IET reduction varied systematically across the diurnal cycle. The largest reductions occurred between 12:00 and 18:00, with mean differences reaching up to 18°C, coinciding with peak regional heat conditions. Smaller IET reductions were observed near sunrise (∼06:00) and during the October measurements (Figure 3, Figure S5 in Supporting Information S1). Only 1.9% IET values exceed regional *T*_db_ suggesting that participants engaged primarily in cool‐seeking behaviors during measurements.

Temporal patterns in IET reduction were further supported by estimated diurnal cycles from the GAM‐IET model predictions (Figure S7 in Supporting Information S1). Consistent with within‐day exposure variability, participants in the *highest overall exposures* profile exhibited smaller reductions relative to regional *T*_db_, with afternoon peak reductions of approximately 12°C. In contrast, participants characterized by *strong diurnal amplitude* of IET showed reduction trajectories that diverged from the full group, with comparatively larger reductions during nighttime hours and greater 24‐hr variability in departure from regional conditions.

In contrast to IET, participants' IEH showed smaller deviations from regional conditions, reflecting the generally dry ambient environment. Differences between IEH and regional *P*_*v*_ were centered near zero (median 0.2 kPa, IQR −0.3 to 0.6 kPa), corresponding to a roughly 9%–18% change in relative humidity (Figure 6d). Across all observations, IEH exceeded regional *P*_*v*_ in 60% of records, indicating a systematic tendency for higher humidity in participants' immediate environments.

### 3.6 Heat Stress Implications of Regional and Personal Heat Exposure

**Figure 7:**
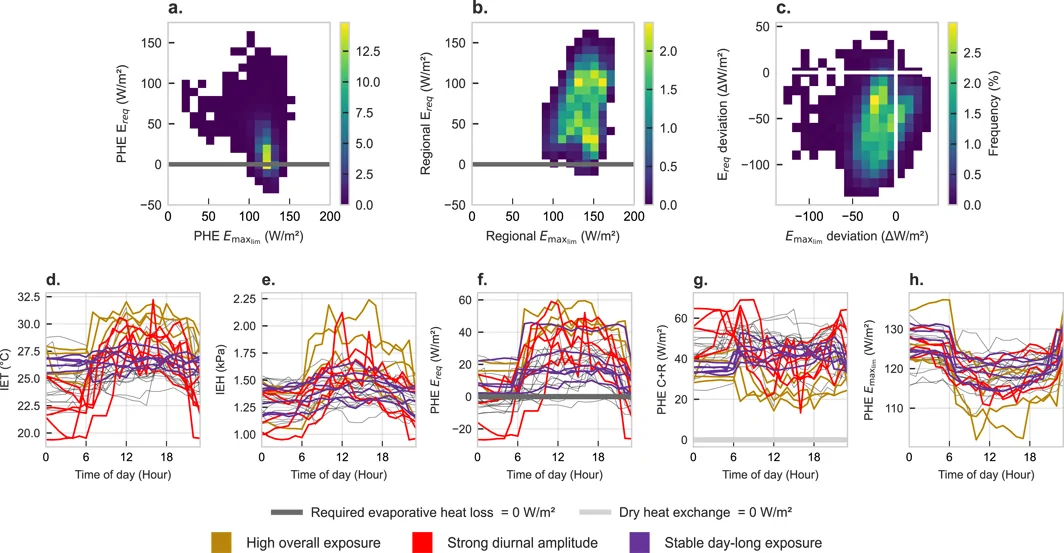
(a–c) 2D histograms indicating required evaporative heat losses to achieve heat balance ($E_{\text{req}}$) (*y*‐axis) and corresponding maximum potential on evaporative loss at skin ($E_{\max_{\lim}}$; $\min(E_{\max,\text{env}};E_{\max,\text{sweat}}$) (*x*‐axis) estimated from 1‐hr observed PHE (a), regional heat (b), and heat differences between PHE and regional heat (c). (d–h) Diurnal cycles of IET (d), IEH (e), $E_{\text{req}}$ (f), dry heat exchange at skin (g), and $E_{\max_{\lim}}$ (h), per participant highlight subjects with high overall exposure (gold), stable day‐long exposure (purple), and strong diurnal amplitude (red). Estimates of $E_{\text{req}}$ were stratified by average awake‐sleep self‐reported periods, with metabolic heat production at *M* = 1.5 MET (sedentary to light activity) for awake and *M* = 1 MET (rest) for sleep. The histograms are divided into bins of 10 W/m^2^, and the gray lines indicate null heat exchanges. All estimates are conservative, as higher activity intensities or additional radiative load (e.g., sun exposure) would increase $E_{\text{req}}$.

Median evaporative requirements to achieve heat balance (*E*_req_) from all participant‐hours from PHE were low —14.5 (2.0–27.5) W/m^2^—upon the highly conservative scenario of participants requiring to compensate for rest (during bedtime) and light activities (awake time) with no external radiation loads (i.e., full shade) and low windspeed. Dry (sensible) heat losses at skin vary around 42.8 (34.2–50.2) W/m^2^, while potential evaporative heat losses ($E_{\max_{\lim}}$) stayed within 121.4 (117.1–150.6) W/m^2^ (Figure 7a). Hence, on average, the metabolic heat generated at rest (1 MET ∼53 W/m^2^) could be dissipated primarily through dry heat loss from the skin. There is also additional heat loss from respiratory fluxes and a greater heat‐loss potential via sweat evaporation at higher activity levels (i.e., greater metabolic heat production). In contrast, if participants were exposed to the regional conditions (i.e., no cool‐seeking behavior), their *E*_req_, on average, would have been 71.8 (43.2–100.3) W/m^2^ with net convective heat gain from the environment (Figure 7b), indicating a greater heat loss reliance on sweating to maintain heat balance.

As a reference and to account for sweating efficiency, the *E*_req_ estimates translate into required sweat rates (*S*_req_) for rest/light activities in the shade of roughly 0.04 (0.01–0.32) l/hr for PHE and 0.21 (0.12–0.32) l/hr for regional conditions. *S*_req_ values related to PHE are extremely low, as they fall within insensible perspiration ranges (i.e., skin diffusion and respiratory tract water loss), with values remaining below 1 l/day (Lamke et al., 1977; Parsons, 2014). Regional average values, despite being above the insensible water loss (20 ml is roughly 1.5 tablespoons of sweat), are not larger overall than sweat rates that are usually achieved during intermittent walking in moderate and warm environments (∼3 METs) (Cramer et al., 2022; Drinkwater et al., 1977).

Likely through cool‐seeking behavior, participants reduced their *E*_req,_ avoiding a heat load of 53.4 (36.4–76) W/m^2^, and shifted sensible heat exchange from convective heat gain to loss (Figures 7c, 7f, and 7g). The largest avoided convective heat gains (∼135 W/m^2^) occurred between 14:00 and 17:00. Conversely, $E_{\max_{\lim}}$ potential was slightly reduced in actual exposures on average by 18.0 (28.7 to −5.7) W/m^2^.

Diurnal patterns in heat exchange further illustrate inter‐individual differences, especially across distinct exposure profiles (Figures 7d–7h). Lower heat stress expressed with the lowest $E_{\text{req}}$ and highest $E_{\max_{\lim}}$ occurred during sleep time. During daytime hours, some participants (mostly those with stable day‐long exposures) exhibited an *E*_req_ closer to zero, indicating a higher capacity to accommodate additional metabolic heat. Participants with higher IETs have higher *E*_req_, but the corresponding average *S*_req_ remains within the insensible perspiration range for this conservative scenario.

## 4 Discussion

This observational study examines inter‐individual variability in temperature and humidity during 24‐hr PHE relative to regional heat, heat‐coping resources, and broad vulnerability. Understanding differences in the ability to avoid the heat and reduce heat stress is critical in regions experiencing sustained, chronic heat, where heat‐related health risks are prevalent, particularly among older adults (Ebi et al., 2020).

This study contributes to the growing body of PHE research in three ways. First, we provide 21 days of 24‐hr PHE data from middle‐aged and older adults in a hot, arid region characterized by persistent extreme heat and high AC prevalence. To the best of our knowledge, this study provides the longest participant‐days reported in a PHE study. Second, we demonstrate that the magnitude and timing of within‐day PHE variability provide critical context for interpreting behavioral heat adaptation beyond daily summary metrics. As PHE data show, a person adjusts to overheated regional conditions through environmental selection or modification. Third, we are the first to translate exposure data into personal heat loads, interpreting potential sensible and evaporative exchanges from actual exposures.

### 4.1 Vulnerability, Adaptive Capacity, and Structural Associations With PHE

We found higher peak daily exposures associated with constrained financial capacity. Specifically, participants earning lower incomes were associated with higher daily IET_max_. Living in a context of high social vulnerability was suggestive of a similar association. We also found pre‐adjusted associations between IET_max_ and limitations in affording AC, and between IET_mean_ with income, but these associations did not survive adjustments for multiple testing. Despite differences in experimental designs, these results and the magnitudes of daily IET_mean_ and IET_max_ are fairly consistent with local studies conducted on diverse adult populations (Hondula et al., 2021; Zhao et al., 2021). For 64 residents (no age specified) exposed for 4 days to an average regional *T*_db_ 32.7°C, Hondula et al. (2021) reported higher IETs (∼1.7°C), lower daytime‐nighttime differences (3.0°C vs. 0.9°C in this study), and IET associations with time spent outdoors, income, and neighborhood. Zhao et al. (2021) recorded 10‐day data from 59 participants reporting average indoor NWS Heat Index (HI) values of 26°C, comparable to the IETs from participants with day‐long stable exposures (i.e., primarily indoors). The same study showed HI consistently >32°C during outdoor exposures, often exceeding 40°C, similar to the maximum hourly IETs above 40°C reported by 30% of participants in the current study. The HI can be compared with the IET due to the close relationship between HI and *T*_db_ in dry environments.

PHE associations with income (and suggestive with social vulnerability) highlight a contextual and structural problem: who can afford to avoid the heat? In this sample of participants with diverse lived experiences, participants within the top 5th median IET earned less than $40,000/year and were exposed to 27–32°C for ∼18 hr/day (Figure 7d). As a reference, these temperatures exceed the regulation for allowable indoor conditions (<27.8°C) required by landlords in Phoenix when an AC system is installed (City of Phoenix, 2015). This group is likely unable to afford AC repairs and is not protected by this regulation (four of the five are homeowners; one participant and the 5th participant are in a living arrangement without paying rent). In addition, two of these participants live in manufactured homes and lease the land, making it harder for them to access assistance (Phillips et al., 2021). Therefore, the lack of statistical effect in PHE from housing features should not be interpreted as a lack of risk, but rather a sample size problem due to high variability in IET, for the following reasons: previous studies found that indoor temperatures are influenced by home size and thermal preferences (Larsen et al., 2022; Wright et al., 2020); living in manufactured homes placed people at disproportionately high heat risk (Charley et al., 2023; Phillips et al., 2021); heat‐vulnerable households may sustain indoor comfort by sacrificing other essential needs (Wright et al., 2020); and no outages that challenge housing thermal resilience were reported during the study (Baniassadi & Sailor, 2018; Rajput et al., 2021). However, this equity gap in heat adaptation requires not only affordable alternatives, but also ensuring they are accessible and widely accepted to avoid unintended consequences reinforcing the gap (Oberai et al., 2026).

### 4.2 Time of Day and Regional Heat as a Standpoint for Interpreting PHE

We also confirm that time of day and regional heat are key determinants of IET, substantiating the findings of Meng et al. (2025). However, PHE deviations from regional heat have primarily been used to quantify exposure misclassifications, yet their value as a metric has not been acknowledged. As baseline climate variability in a place sets the need to adapt to warm/hot/cold or dry/humid conditions for short or long periods, the actual magnitude of heat deviation (i.e., ΔIET = personal − regional) then informs how much people modify their environment to support individual adaptation/maladaptation processes. Time of day is also an important factor for relating PHE data to behavioral adaptation, since weather, circadian rhythms, and personal routines are shaped by 24‐hr cycles. Consistent with previous studies examining hourly‐scale temporal variations (Hass et al., 2022; Meng et al., 2025), we confirm that IET maximums/minimums do not consistently occur during traditional daytime/nighttime hours.

The current study was conducted during the hottest summer on record in Phoenix, and, on average, IET was reduced by 10.4°C relative to regional *T*_db_, reaching up to 18°C during the afternoon (Figures S5 and S7 in Supporting Information S1). This elevated value highlights the challenge of managing sub‐diurnal peaks in electrical demand to keep AC running (Ruddell et al., 2014). Other studies in mid‐latitude cities with high presence of AC but lower regional air temperatures report average IETs reductions within 2–4°C but do not report the inter‐diurnal variability that shows IET reductions up to ∼8–11°C during a heat‐wave (E. R. Kuras & Hondula, 2015) and for urban residents (Bernhard et al., 2015). Conversely, positive ΔIETs within ∼+2–4°C have arisen in places with expected moderate summers and in occupational settings in hot‐humid settings (Butler‐Dawson et al., 2024; Han et al., 2025; Milando et al., 2022). For these studies, IET > regional *T*_db_ levels were explained by nighttime indoor heat retention, AC failing to provide desired thermostat levels, and/or active outdoor work. Finally, seasonal variation can be further explored since systematic biases from ΔIETs have been found in a well‐heated U.S city with cold winters and warm summers (Meng et al., 2025), but not in rural areas with tropical climate where AC is not spread (Deshpande et al., 2024). Overall, ΔIETs relative to a baseline regional *T*_db_ reflect differential pressures on people to adapt at different time scales (few days, months, or even all‐year‐long), the broader use of cooling infrastructure (whether generalized, adopted, or under‐ or well‐dimensioned), and adjustments in daily activities.

### 4.3 Biophysical Interpretation From PHE

Heat stress is a critical step in the causal pathway linking environmental exposure to heat‐adverse health outcomes, a factor often, if not always, missed in PHE research (Guzman‐Echavarria et al., 2022). Some studies assess PHE using the NWS HI (e.g., Sugg et al., 2022), but the HI does not account for specific variations in activity, clothing, wind, or radiation, or specific biophysical features of individuals. Therefore, we use a human‐environment heat‐exchange approach rather than a single heat‐stress index to assess personal heat stress. This approach enables showing heat stress in terms of thermoregulatory demands/capacities when direct strain measurements are unavailable (e.g., core and skin temperature, heat perception, sweating, heart rate, skin blood flow), as there is ample evidence that *E*_req_ drives whole‐body sweat rate upon compensable heat stress (Gagnon et al., 2013). Our results indicate that, at rest or during light‐sedentary activity, most PHE participant‐hours require minimal thermoregulatory demand (i.e., with likely low heat strain), as *E*_req_ remained within ±15 W/m^2^ during 48.8% of the hours. Therefore, metabolic heat could be dissipated primarily through sensible heat loss, and *S*_req_ would remain within the range of insensible perspiration rates. In contrast, participants with wide diurnal amplitudes and higher sustained exposures (Figure 7f) exhibited reduced convective heat loss during the daytime (i.e., higher *E*_req_), suggesting evaporative cooling requirements and a greater potential need for active thermoregulatory responses, such as vasodilation and sweating. Notice that evaporative heat‐loss potential from sweating is generally sufficient to maintain compensable heat stress (*E*_max_ > *E*_req_), indicating that higher activity levels and moderate radiation loads could be accommodated under most PHEs found here. In contrast, if participants were continuously exposed to regional conditions, as many epidemiological studies do, the heat‐stress risk would be substantially higher due to exposure misclassification. Under this non‐action scenario, low *E*_req_ within ±15 W/m^2^ would only happen 3.8% of the time, and participants would even gain sensible heat from the environment (i.e., dry heat exchange < 0) during 61.1% of the time (vs. 1.4% based on PHE).

This biophysical analysis has inherent uncertainties because the estimates are conservative due to unmeasured attributes. We assumed 24 hr with rest or sedentary activities; light clothing; no external radiation loads; low wind speed; and similar sweating capacity and body composition across participants; all factors affecting heat exchange (Cramer et al., 2022). We ran the analysis within common ranges of wind speed and maximal sweat rate (Figures S8 and S9 in Supporting Information S1), and the interpretation of the results remains stable. Therefore, we only report 0.3 m/s results as the most conservative condition. Also, since real‐world exposures are likely to be transient for much of the time, these results should be interpreted with caution, as thermal responses may lag rapid environmental transitions (Stolwijk & Hardy, 1966). Still, only 10.4% of participant‐hour IET fluctuate IET by >±5°C within 1 hr, and removing those records from the analysis would only shift the *E*_req_ and *E*_max_ medians by 2 and 0.2 W/m^2^, respectively. A better representation of transient exposures can be achieved using detailed strain models once they are validated in natural settings.

Because most studies on physiological heat response are conducted in controlled indoor environments, information from natural settings can help other disciplines extend their applications to more realistic conditions (Vecellio & Vanos, 2024). For example, thermal physiologists could use personal heat‐exchange estimates in experimental designs to explain how real‐world behavior protects or harms people in the heat. Whereas built‐environment researchers could incorporate such data to refine occupancy assumptions and personal thermal comfort models (Arakawa Martins et al., 2022). Overall, our results demonstrate how inter‐individual 24‐hr variation in PHE can be translated into personal heat loads to inform which activity spaces and for whom should be prioritized in heat mitigation strategies.

### 4.4 Advancing PHE Research for Heat Adaptation and Resilience

Given that heat adaptation goals and needs vary across places and cultures, and that adaptation indicators are undermined at the household and hyperlocal scale (Loroño‐Leturiondo et al., 2025), context‐specific PHE data are essential for tailoring local adaptation. Future PHE studies will continue to expand global representation and strengthen links between heat mitigation and adaptation strategies to everyday thermal experience. Broader community‐wide and occupational data collection supports the identification and description of heat‐vulnerable groups, increases self‐awareness on heat safety, clarifies decision‐making related to relief and cool‐seeking behaviors (e.g., proactive vs. reactive) and their effectiveness. For example, partnerships with non‐academic actors have shown the potential to co‐produce context‐specific knowledge, making explicit the everyday burden of heat from housing and providing specific focal action points (Phillips et al., 2021; Vant‐Hull et al., 2018). Simultaneously, methodological advances are needed to address challenges in behavioral data collection (see discussion in Zhao et al. (2021)) (e.g., EMA, GPS‐based tracking, automated sensing) and analysis (e.g., causal inference, mediation, spatiotemporal analysis) to better link specific behaviors and physiological outcomes to specific heat loads. Finally, observed relationships between PHE and regional weather suggest opportunities to develop probabilistic PHE forecasts calibrated to local climate and sub‐population vulnerability/adaptive characteristics, enabling more targeted and effective heat early‐warning systems.

### 4.5 Study Limitations

This study has several measurement and analytical limitations that should be considered when interpreting the results. The participant sample size was small for determining statistical significance (yet on par with, or higher than, related PHE studies and with greater temporal coverage). The study is also biased toward the heat‐aware population, as recruitment included partners from resilience initiatives and resulted in enrollment of highly motivated participants who were not concerned about multiple in‐home sensors or the burden of participation over 3 weeks. We relied on participants consistently carrying wearable sensors, which added uncertainty, yet was mitigated by applying extensive data‐matching procedures, automatic and supervised at the participant‐day level. Wearable measurements are also subject to biases related to direct solar radiation, highly impervious environments, and on‐body placement. However, the Kestrel Drop D2 is less sensitive to these effects (Bailey et al., 2020). From an analytical perspective, the relatively small sample size restricted stratification across socioeconomic and demographic groups, and the lack of continuous activity tracking limited inference about specific adaptive behaviors. Many exposure metrics do not follow normal distributions, necessitating flexible statistical approaches; we addressed this issue by using GAMs. Using a single fixed‐weather station to represent a baseline regional weather signal may contain a geographic bias (i.e., altitudinal) unrelated to intra‐urban thermal variations conditioned by urban design and socio‐economic factors. Participants' residences were at similar altitudes and far from complex terrain features. Finally, in the heat stress analysis, we were unable to account for concurrent, unmeasured variations in radiation, wind, metabolic activity, and clothing that can affect the overall heat load.

## 5 Conclusions

This observational study assessed intra‐ and inter‐individual variation in PHE and heat loads for middle‐aged and older adults across the hot, dry Phoenix Metropolitan Area. Results demonstrate that 24‐hr PHE data provide a high level of detail for *interpreting and understanding* heat adaptation in a place with chronic heat and high AC prevalence. Overall, regional air temperatures are poor predictors of actual heat exposure, but provide a meaningful signal of behavioral adaptation when contrasted with actual exposures, as participants reduced their IET by 10.4°C on average (reaching 18°C in the afternoon hours) across the 4‐month study period. Despite finding exposures that would lead to low heat stress for sedentary activities on average, we observed participants who deviated from the sample‐average pattern due to higher sustained 24‐hr exposures (i.e., 29–32°C for 18 hr/day) and greater diurnal PHE variability. Ultimately, those participants would also experience greater physiological heat stress (and likely higher strain) when deviations from this study's assumptions occur.

Inter‐individual differences in heat avoidance (ΔIET) reveal who can better avoid heat and their implications for everyday heat stress. As growing evidence shows, we also reinstate the affordability bias in heat adaptation, demonstrated here by middle‐aged and older adults with diverse lived experiences, vulnerabilities, and daily routines during a record‐breaking Phoenix summer. Participants earning less than $40 K/year experienced significantly greater heat exposure than those earning more. Suggestive and pre‐adjusted PHE associations with social vulnerability and economic trade‐offs to afford AC are consistent with that finding.

Overall, our findings demonstrate that high‐resolution personal heat monitoring captures behavioral adaptation to heat through environmental selection and modification. This information provides critical insights into heat‐coping strategies at the individual activity‐space level, relying on the granularity of ancillary data. Such an ecologically valid understanding of heat exposures in free‐living conditions can guide both person‐centered and population‐level strategies for heat adaptation. Highlighting when and why interventions may be most effective to further inform strategies at different governance levels using locally grounded evidence.

## Conflict of Interest

The authors declare no conflicts of interest relevant to this study.

## Supporting information

Supporting Information S1

## Data Availability

The personal heat exposure, physiological, perceptual, and survey data supporting this study are archived in Environmental Data Initiative under “GCHA HeatSuite Phoenix 2024 pilot: personal heat exposure, physiological and perceptual responses in a group of older adults,” available at Guzman‐Echavarria et al. (2026) under a CC0‐1.0 license. Analysis and visualization codes (R and Python) to reproduce all figures and tables are available at Guzman‐Echavarria and Caldwell (2026) (Version 1.1.0). GCHA and publications are also registered within the Open Science Method (OSM) framework (Gagnon, Vanos, et al., 2026).
