# Supplementary material for: Who Can Avoid the Heat? Inter‐Individual Differences in 24‐hr Heat Exposure Reveal Disparities in Heat Adaptation and Stress
Source: Geohealth. 2026 Sep 12;10(9):e2026GH001914. doi: 10.1029/2026GH001914 (PMC13570422; doi:10.1029/2026GH001914)
Supplement: Supplementary file 1 [file GH2-10-e2026GH001914-s001.docx]

*GeoHealth*

Supporting Information for

**Who Can Avoid the Heat? Inter-individual Differences in 24-h Heat Exposure Reveal Disparities in Heat Adaptation and Stress**

G. Guzman-Echavarria^1^, A. Middel^1^,^2^, N. Ravanelli^3^, J.B. Rosales Chavez^1^, A.R. Caldwell^4^, C. Metzler^1^, K. Rentz^1^, M. Munoz^5^, M. Guardaro^5^, and J.K. Vanos^5^.

^1^School of Geographical Sciences and Urban Planning, Arizona State University, Tempe, AZ 85287-5302, USA.
^2^The GAME School, Arizona State University, Tempe, AZ 85287-5802, USA.
^3^Department of Physiology, National University of Singapore, 117593, Singapore
^4^Department of Biostatistics, Fay W. Boozman College of Public Health, University of Arkansas for Medical Sciences Northwest, Springdale, AR 72762, USA
^5^School of Sustainability, Arizona State University, Tempe, AZ 85287-7904, USA

**Contents of this file**

Text S1 to S6

Figures S1 to S9

Tables S1 to S3

**Introduction**

This supporting information contains material that complements the methodological design of the study, including participant recruitment, data collection, pre-processing, and details on statistical model implementation. It also contains complementary results through figures showing details on individually experienced humidity and personal thermal comfort by participants, and sub-diurnal details by participant that were not shown (but described) in the main manuscript.

Text S1. Participant Recruitment and Eligibility

The recruitment process involved simultaneous outreach strategies and the distribution of a short intake survey via REDCap (Harris et al., 2019) to screen eligibility, followed by a first contact via email or phone to confirm availability and schedule the first in-person visit. The inclusion criteria were age ≥45 years, residing in single-unit, multi-unit, or manufactured homes, staying in the local area during the summer of 2024, and being able to read and understand English or Spanish. Potential participants who were unable to consent, required third-party consent, or suggested hazardous or harmful alcohol consumption were excluded. Constrained by the availability of 10 HeatSuite units, we did not aim to enroll a population-representative sample of the older population, but rather to capture diverse lived experiences across socioeconomic positions.

Recruitment occurred from May to October of 2024 with a mix of referrals and neighbors (snowball sampling) from different concurrent outreach strategies to guarantee participant intake from a diversity of living experiences (i.e. a wide spectrum of socio-economic conditions). Recruitment strategies comprised flyers and personal communication and/or community events with various collaborators (i.e. ASU Knowledge Exchange for Resilience, Mesa Center for the Arts, Arizona Association of Mobile Home Owners - AAMHO, CHISPA AZ, posting flyers in public libraries and senior centers, ASU HeatReady website, and sending information via email list servers from ASU School of Sustainability). Recruitment initially began with adults aged 65 years or older, but later expanded to include those aged 45 years or older to increase enrollment. Participants were enrolled into one of five data collection waves based on the timing of recruitment and participant availability

Initial enrollment included 65 individuals who completed the intake survey, but 39 were retained completed the study. Reasons for not continuing participation included: incomplete contact information; participants under 45 filling out the intake survey; non-response to our calls, emails, or text messages to schedule visits; and a decline to participate after learning further information about the study. For example, other people in the home feel uncomfortable about having sensors installed.

Text S2. Study Procedures and Personal Heat Exposure Data Collection

First contact with participants occurred during recruitment and finished when we provided them with an interpreted report of their data (Refer to Fig. 1 and 2 in the main manuscript for the full timeline). Recruitment began with flyer distribution and participation in community events, during which participants completed a web-based intake survey. Additionally, participants made many referrals during the study. Later, the research team communicated with potential participants after screening, via cellphone or email (as they preferred), to schedule an initial in-person visit. During this visit (~60-90 minutes), participants provided written informed consent, completed a baseline survey via REDCap, received all monitoring equipment, and were informed of the sensor-use protocol. They received a full HeatSuite kit (Ravanelli et al., 2025) (including an environmental node, a Bangle.js 2 smartwatch, a blood pressure cuff, and an oral temperature probe) and 3 Kestrel drops sampling every 10 minutes. For this research study, we used Bluetooth Low Energy (BLE) communication between the environmental node and the smartwatch (both parts of the HeatSuite system) to infer the participant’s presence at home. Further data from the HeatSuite is being used in future studies. Thus, we rely on environmental data (personal exposures) from the Kestrel Drops. One Kestrel drop was placed in the more frequently used room (i.e., living room), right next to the HeatSuite environmental node. A second Kestrel drop was placed in the participant's bedroom, and participants carried a third on their bodies to capture outdoor exposures or exposures outside of the home. We verified that at-home sensors were placed far from windows to avoid sun exposure, away from direct sources of heating, such as the kitchen, and not in the direct path of air conditioning (AC) outlets. The Kestrel D2 sensors have accuracies of ±0.5 °C and ±2 % for temperature and relative humidity, respectively, and exhibit the least bias among the commercial sensors commonly used in PHE studies (Bailey et al., 2020; Habeeb et al., 2022).

After that first visit, each participant began a 21-day data-collection period during which they wore a Kestrel drop attached to their bag or belt loop using a carabiner when they left home. Researchers occasionally contacted participants to troubleshoot equipment issues, conduct wellness checks (if indoor temperatures exceeded 35 °C while a participant was at home), or to provide clarification on the protocol as needed. Some troubleshooting also involved in-person visits. At the end of the 21 days, a second in-person visit (~60-90 minutes) was held to complete an exit survey via REDCap and retrieve equipment. During the last visit, we shared each participant's temperature data from 3 Kestrel drops and discussed it with the participant. We also prepared a report summarizing their data and sent it once the data screening process was complete. Each participant contributed about 14 hours of active engagement to complete the study from beginning to end, including visit times, environmental exposure data, and all the interactions with HeatSuite equipment to gather heat perception and physiological records. Overall, a person would take 5 to 10 minutes to complete their records (3 times a day), including the time needed to rest before measuring their blood pressure. If we count only the time spent taking exposure records (no other HeatSuite tasks), it would not exceed 4 hours of active engagement, including initial and end visits, plus few minutes to remember to take the Kestrel with them.

Each participant received appropriate economic compensation for volunteering their time.

Text S3. Personal Heat Exposure Reconstruction Procedure

To reconstruct personal heat exposure (PHE) from each participant, we had data for three Individually Experienced Temperature (IET) and humidity (IEH) time series as input from 1) the living room, 2) the bedroom, and 3) on-body, plus BLE proximity data between the HeatSuite environmental node and smartwatch. Therefore, we designed a two-step process to reconstruct a unique PHE time series for each person by selecting the sensor that best represents the participant's exposure for each 10-minute interval (Figures S1 and S2). We first applied an automatic reconstruction process, and later (using time series plots), manually screened each participant-day dataset for implausible activity patterns indicating non-compliance with the study design, as well as for participant-days with missing BLE data.

We designed an automated PHE reconstruction process (Figure S1) to infer which sensor most likely represented the participant's location at each 10-minute timestamp. Classification criteria were based on self-reported wake-up and bedtime, and BLE as a proxy for detecting when participants were “at home”. We stated that a participant was at home if there was at least one BLE proximity signal between the node and the smartwatch during the 10 minutes before the kestrel timestamp, since the watch and node communicate every 5 minutes. To assign the most likely sensor for each timestamp, we first determined whether the timestamp falls within bedtime or awake hours, based on each participant’s self-reported bedtime and wake-up times. For participants who did not report their wake-up and bedtime hours (10 out of 34 participants), the default awake time was set to 7:00 AM to 11:00 PM. At bedtime hours, bedroom PHE data were assigned. During wake hours, on-body data were assigned by default, but were overridden with living room data when the participant was at home.

Figure S1. Criteria for automatic personal heat exposure reconstruction time series from Kestrel Drop data, based on BLE data and participant self-reported usual times to go to bed and wake up.

This approach generally produced reliable results, and we found it beneficial to be able to compare two sensors during daytime conditions since the living-room sensor was not exposed to sunlight, thus allowing us to mitigate the reported overheating bias in Kestrel sensors, which may persist for over 30 minutes after sun-exposed conditions (Habeeb et al., 2022).

However, for participants living in manufactured homes, BLE signals indicated that participants were at home most of the time, even when substantial differences existed between on-body and living-room records. Thus, we used the wearable (on-body) sensor as the daytime primary exposure sensor (and did not use living room data) to avoid likely underrepresentation of outdoor exposure relative to indoor exposure compared to other housing types. We hypothesized that BLE-based “at home” over-detection reflects the small size and thin walls typical of manufactured homes. This decision was made with consideration of the fact that all participants living in manufactured homes demonstrated high protocol compliance.

As a second step, after the automatic process, we manually screened records that indicated implausible activity patterns or suggested non-compliance with the study protocol. This review was conducted independently by two researchers, who separately reviewed suspicious participant-days (i.e., 84 participant-days) to reach consensus on whether to adopt automatic reconstruction, use only wearable data, use only living room data, or exclude the participant day altogether. As a result, two participants (ID: 07 and 23) were removed from the analysis due to unrealistic IET patterns.

Figure S2. Example of personal heat exposure reconstruction for one participant, showing raw data from three Kestrel Drop sensors and BLE-based “at-home” detection information, along with the intermediate data used to assign the most representative sensor to each 10-minute timestamp. The criteria for automatic reconstruction are in Figure S1.

Text S4. Daily PHE metrics time series: redundancy analysis and inter-individual variability

For the PHE metrics time-series analysis, we derived daily-aggregated PHE metrics following Hondula et al. (2021) to characterize exposure magnitude, duration, and frequency. We additionally incorporated variability metrics (e.g., variance and diurnal range) to capture the spread of thermal conditions experienced during daytime and nighttime periods (Table S1). To address potential autocorrelation among metrics and reduce the large number of permutations from the PHE metric and adaptive capacity factors, we performed a redundancy analysis using a correlation matrix (Figure S3).

| **PHE metric** | **PHE dimension/aspect** | **Definition** |
| --- | --- | --- |
| **Mean IET/IEH** | Intensity - central tendency | Magnitude of IET/IEH on average |
| **Max IET/IEH** | Intensity - extremes | Magnitude of IET/IEH during the hottest hour (daytime peak exposure) |
| **Min IET/IEH** | Intensity - average minimum | Magnitude of IET/IEH during the coldest hour (nighttime lowest exposure) |
| **Mean daytime/nighttime IET/IEH** | Intensity - central tendency daytime/nighttime | Magnitude of IET/IEH during daytime and nighttime based on sunlight hours |
| **Variance daytime/nighttime IET/IEH** | Intensity - variability daytime/nighttime | Spread of IET/IEH during daytime and nighttime based on sunlight hours |
| **Daily range IET/IEH** | Intensity - amplitude | Daily range of IET/IEH as the daily maximum minus the daily minimum (amplitude of heat experienced) |
| **Longest Exposure Period (LEP) IET**** | Duration | Greatest number of continuous minutes with IET above threshold* |
| **Degree Minutes above Threshold** (DMAT) IET** | Frequency & Intensity | Sum of heat observations (in minutes) exceeding the threshold* scaled to adjust for missing observations |
| **Percentage Minutes Above Threshold** (PMAT) IET** | Frequency & Intensity | Relative exposure burden % - Ratio of IET observations above threshold* to total number of observations |
| IET: Individually experienced temperature /IEH experienced humidity  *We used a fixed IET threshold of 24°C to hypothesize an upper threshold for usual thermoneutral conditions and achievement of thermal comfort. | | |

Table S1. Daily personal heat exposure metrics based on IET and IEH in this study.

Overall, there was no strong correlation between IET and IEH metrics, but within IET and IEH groups, three types of high correlation emerged with R2>0.6 (Figure SM3).

**Strongly related (R² 0.6–0.8):** Cluster1: Maximum, daytime variance, and daily range. Cluster 2: Minimum, nighttime mean, proportion time above 24°C.

**Redundant (R2>0.8):** Cluster 3: Mean, daytime mean, degree-minutes above 24°C (notice that threshold chosen was close to the dataset’s central tendency).

Figure S3. Hierarchically clustered heatmap displaying a correlation matrix from IET-based PHE daily metrics.

Overall, there was no strong correlation between IET and IEH metrics, but within IET and IEH groups, three types of high correlation emerged with R2>0.6 (Figure SM3).

Later, we applied linear mixed-effects models to predict each metric with a fixed effect for mean daily regional temperature and a random intercept for each participant (see Section SM6 for model details). Across clustered groups of variables, only daily IET_min_, IET_mean_, and IET_max_ showed linearity, and we retained them for further analysis.

It is worth mentioning that daily PHE metrics exhibited intraclass correlation coefficients (ICCs) ranging from 0.21 to 0.82, indicating substantial variation in the extent to which metrics capture participant-specific versus regionally driven variability. Metrics with lower ICCs (e.g., variance and daily range) more closely tracked day-to-day fluctuations in regional weather, whereas metrics with higher ICCs (e.g., nighttime magnitudes and IET_mean_) were more strongly structured by participant-specific context.

Text S5. List and Inclusion Criteria of Vulnerability, Adaptive Capacity and Sensitivity Factors Considered in this Study

Entry and exit surveys allow us to gather rich multi-dimensional information about the type and level of resources that each participant has access to cope with general stressors that are specific to heat. . Also, we collected some factors related to sensitivity (i.e., physiological or social characteristics that make an individual more "susceptible to injury), and extracted a comprehensive metric based on the participants’ house location a comprehensive metric related to their overall social vulnerability. We also asked participants for proxies of indoor thermal exposure related to housing characteristics, AC type and use, thermostat settings/preferences, and usual indoor occupancy times.

To focus our analysis, We first select variables found to be relevant to differentiating heat exposure and indoor temperatures in the region (Hondula et al., 2021; Larsen et al., 2022; Wright et al., 2020). Additionally, we added age, heat-coping narrative (Guardaro et al., 2022), and heat-risk comorbidities to the list of variables. Furthermore, we applied redundancy and clustering analyses to retain predictors providing complementary information. The redundancy analysis identified variables other variables in the dataset could reasonably predict. As a result, thermostat settings, preferred temperatures, number of rooms, and economic trade-offs to keep the AC running were found to be predicted by the other variables at a cut off R^2^=0.5 and were therefore removed from the analysis with minimal information loss. At a later step, after finding associations between daily peak exposures and income, we decided to include “limitation to afford AC” back into the analysis as a direct measure of heat-adaptation affordability. As a confirmatory step, we applied a Hoeffding's D clustering finding that all factors were highly unrelated. These analysis were done using redun and varclus functions from Hmisc R package.

In Table S2, we present the full list of variables,their respective recoding procedures and associated concepts (e.g., vulnerability, adaptive capacity or sensitivity). For recoding, given the small sample, we prioritize evaluating dichotomous variables and equally sized sub-groups.

| **Factor (type, included in analysis)** | **Question in survey** | **Type of variable and processing method** | **Frequency:  count (%) or mean (std)** |
| --- | --- | --- | --- |
| ****Housing type** (Adaptive capacity, **included**) | res_type | Categorical (recoded to retain 3 categories): (1) Single-family home detached/attached, (2) Apartment in 2-4 stories building, (3) Manufactured home. | Single-family home detached/attached 22 (64.7), Apartment in 2-4 stories building 4 (11.8), Manufactured home 8 (23.5) |
| **** Number of bedrooms - building size proxy** (adaptive capacity, **non-included**) | n_rooms | Open-ended numerical (range: 1-11). (Recoded from survey questions: “b_bed” + “n_other_rooms”) | 5.14 ± 2.19 |
| **Central AC** (adaptive capacity, **included**) | ac_central | 0 (No) or 1 (Yes) (recoded to restrict to a dichotomous variable)  (1) Central air conditioner or heat pump, (0) mini split, window or wall air conditioner, evaporative or swamp cooler. | Yes 27 (79.4), No (including wall/window AC, mini-splits, and evaporative (swamp) cooler) 7 (20.6) |
| **** Limitations on AC use during data collection**  (adaptive capacity, **included**) | lim_ac_use | 0 (No) or 1 (Yes) (recoded to express when a participant has access to AC AND limits the use during measurements) | Yes 3 (88.2), No 30 (8.8), NA 1 (2.9) |
| **Fan use**  (adaptive capacity, **included**) | cool_str_ind___1 | 0 (No) or 1 (Yes) (during measurements) | Yes 28 (82.4), No 6 (17.6) |
| **** Set temperature when awake at home**  (adaptive capacity, **non-included**) | ther_day_post | Categorical 9-point scale in 2°C increments from 1 (below 18°C) to 9 (32°C and above) | <18°C (0, 0.0%), 18–20°C (0, 0.0%), 20–22°C (2, 5.9%), 22–24°C (6, 17.6%), 24–26°C (17, 50.0%), 26–28°C (6, 17.6%), 28–30°C (3, 8.8%), 30–32°C (0, 0.0%), ≥32°C (0, 0.0%) |
| **** Set temperature when asleep at home**  (adaptive capacity, **non-included**) | ther_night_post | Categorical 9-point scale in 2°C increments from 1 (below 18°C) to 9 (32°C and above) | <18°C (0, 0.0%), 18–20°C (0, 0.0%), 20–22°C (4, 11.8%), 22–24°C (9, 26.5%), 24–26°C (15, 44.1%), 26–28°C (4, 11.8%), 28–30°C (2, 5.9%), 30–32°C (0, 0.0%), ≥32°C (0, 0.0%) |
| **** Ideal temperature**  (adaptive capacity, **non-included**) | pref_temp_post | Categorical 9-point scale in 2°C increments from 1 (below 18°C) to 9 (32°C and above) | <18°C (0, 0.0%), 18–20°C (1, 2.9%), 20–22°C (1, 2.9%), 22–24°C (5, 14.7%), 24–26°C (22, 64.7%), 26–28°C (4, 11.8%), 28–30°C (1, 2.9%), 30–32°C (0, 0.0%), ≥32°C (0, 0.0%) |
| **Heat coping narrative**  (adaptive capacity, **included**) | self_hadap_post | Categorical (3 categories): | Heat is an inconvenience 26 (76.5), Heat is a manageable problem 5 (14.7), Heat is a disaster or a catastrophe 3 (8.8) |
| **** Home ownership**  (adaptive capacity, **included**) | home_own | 0 (No) or 1 (Yes) (recoded from survey to restrict to a dichotomy variable) | Yes 24 (70.6), No (including paying rent and other occupation types) 10 (29.4) |
| **Limitation to afford AC**  (adaptive capacity, **included**) | tradeoff_spend, tradeoff_spend_ac | 0 (No) or 1 (Yes) (recoded from survey to express when a participant makes difficult decisions to afford essentials during heat to AC, AND limits the use during measurements) | Yes 9 (26.5), No 25 (73.5) |
| **** Main activity outdoors**  (Sensitivity, **included**) | occupation | 0 (No) or 1 (Yes) (recoded to be dichotomous if the participant reported their main activity to be outdoors) | Yes 23 (67.6), No 10 (29.4), NA 1 (2.9) |
| **Age**  (Sensitivity, **included**) | age_est | Open-ended numeric | 59.7 ± 7.90 |
| ****Income**  (adaptive capacity, **included**) | income | Categorical (3 categories) (recoded to obtain equally sized groups) (1) Lower income (less than 40K/year), (2) Average income (40-80k/year), (3) Higher income (more than 80K/year) | Less than 40K/year 12 (35.3), 40-80k/year 11 (32.4), More than 80K/year 10 (29.4), NA 1 (2.9) |
| **Comorbidities**  (Sensitivity, **included**) | health_cond or personal_med_history | (No) or 1 (Yes) (recoded to be dichotomous if any of the following group of illnesses were reported: hypertension, diabetes, stroke/CVA, cardiovascular, respiratory, or kidney disease. | Yes 19 (55.9), No 15 (44.1) |
| **Arizona Social Vulnerability Index (AZSVI)**  (Vulnerability, **included**) | Not in survey, retrieved from AZSVI dashboard*** |  | 0.539 ± 0.24 |
| NA stands for missing data or the participant’s non-answer.  ******These factors were found to be significant associations with indoor temperatures or PHE in Phoenix in previous studies (Hondula et al., 2021; Larsen et al., 2022; Wright et al., 2020).  *** AZSVI dashboard: https://storymaps.arcgis.com/stories/4d003f3686be44cfb846ba5362d133bb | | | |

Table S2. Full list of factors, including specific adaptive factors to heat, included in the study to relate to daily aggregated PHE metrics.

Text S6. Statistical Modeling Framework for Estimating Adjusted PHE Effects

To make a fair comparison from PHE exposure data taken in different months (thus, upon differential regional heat) and account for the hierarchical nature of the dataset (i.e., high autocorrelation within participant data), we used statistical modelling on IET, ∆IET (i.e., IET – regional ambient temperature), and daily PHE metrics to:

- Characterize how much variability in the dataset comes from participant-specific differences (i.e., estimation of the Intraclass coefficient).

- Obtain adjusted predictions upon the same regional weather forcing across participants.

- Estimate sample-level effects of regional temperature, time of day, and individual adaptive capacity.

As a result, we fit two different models:

a) Generalized additive model (GAM) for IET to examine inter-individual differences in personal heat exposures,

b) Nested Linear Mixed Model (LMM) to identify inter-individual differences in PHE dimensions and evaluate whether adaptive capacity factors significantly influenced them.

Model formulations are provided below, with R implementations available in (Guzman-Echavarria & Caldwell, 2026)

a) **GAM model for IET to examine inter-individual differences in personal heat exposures:**

$${IET}_{ij}= \beta_{0}+ f_{1}\left( {Regional\_T\mathrm{db}}_{i} \right)+f_{1j}\left( {Regional\_T\mathrm{db}}_{i} \right)+f_{2}\left( {tod}_{i} \right){+ f}_{2j}\left( {tod}_{i} \right)+b_{j}+\varepsilon_{ij}$$

Where:

*i* = observation (1-h unique record), and *j* = participant (from 1 to 34)

$\beta_{0}$ is the parametric intercept coefficient

$f_{1}\left( {Regional\_T\mathrm{db}}_{i} \right)$ is the population-level thin plate regression spline for regional temperature

$f_{1j}\left( {Regional\_T\mathrm{db}}_{i} \right) \sim GP(0, \tau₁^{2})$ are subject-specific smooth deviations from $f_{1}$. This indicates that each subject-specific smooth deviation is drawn from a Gaussian process with mean zero and a covariance structure scaled by $\tau₁^{2}$ . In practical terms, the function values at any finite set of input points follow a multivariate normal distribution, with the correlation between points determined by their proximity in the input space (here, regional temperature).

$f_{2}\left( {tod}_{i} \right)$is the population-level cyclic cubic spline for time of day (24-h cycle).

$f_{1j}\left( \mathrm{tod}_{i} \right) \sim GP(0, \tau₁^{2})$ are subject-specific smooth deviations from $f_{2}$.

$b_{j}\sim N(0,{\sigma_{b}}^{2})$ are random intercepts for participants.

$\varepsilon_{ij} \sim N(0,\sigma^{2})$ are the residual errors.

b) **Nested LMM to identify inter-individual differences in PHE dimensions and evaluate whether vulnerability factors significantly influenced them.**

We used LMM models, first, to determine inter-individual differences in PHE dimensions and check linearity in metrics (see S4 text), and second, to extend the model and apply it to a reduced list of PHE metrics (3 metrics: IET_min_, IET_mean_, and IET_max_ ) and adaptive factors (11 factors: see section 2.3.3 and Table 1 in the main text).

**First LMM model: Daily PHE metrics with regional temperature as a predictor and random intercept by participant**

$$Daily\_\text{PHE\_metric}_{ij}=\beta_{0}+\beta_{1}{\text{(}Regional\_T\mathrm{db}}_{ij})+b_{j}+\varepsilon_{ij}$$

Where:

$\beta_{0}$ is the fixed intercept (population-average daily PHE metric at mean daily regional temperature),

$\beta_{1}$is the fixed effect centered at the mean daily regional temperature, representing the expected change in the daily PHE metric per 1°C increase in regional temperature.

$b_{j}\sim N\left( 0,{\sigma_{b}}^{2} \right)$ are random intercepts for subjects, capturing stable between-participant differences in daily PHE metrics

$\varepsilon_{ij} \sim N(0,\sigma^{2})$ is the residual error

**Second LMM: Daily PHE metrics with regional temperature and one fixed adaptive factor as predictors and random intercept by participant**

For models including adaptive capacity factors, the model extends to:

$$Daily\_\text{PHE\_metric}_{ij}=\beta_{0}+\beta_{1}{\text{(}\mathrm{Regional}_{T\mathrm{db}}}_{ij})+\beta_{2}(\text{adaptive\_factor}_{j})+b_{j}+\varepsilon_{ij}$$

Where $\beta_{2}$ represents the fixed effect of the adaptive capacity factor. For categorical factors, $\beta_{2}$ yields group-level contrast in the daily PHE metric; for continuous factors, $\beta_{2}$represents the expected change in the daily PHE metric per unit increase in the factor.

| **PHE Metric** | **Vulnerability Factor** | **Model term** | **Wald χ²** | **df** | **Raw  p-value** | **FDR-adjusted  q-value** |
| --- | --- | --- | --- | --- | --- | --- |
| mean_IET | res_type | res_type | 1.959 | 2 | 0.376 | 0.739 |
| mean_IET | ac_central | ac_central | 0.534 | 1 | 0.465 | 0.739 |
| mean_IET | lim_ac_use | lim_ac_use | 3.670 | 1 | 0.055 | 0.378 |
| mean_IET | fan_use | fan_use | 1.351 | 1 | 0.245 | 0.674 |
| mean_IET | self_hadap | self_hadap | 2.007 | 2 | 0.367 | 0.739 |
| mean_IET | home_own | home_own | 0.004 | 1 | 0.947 | 0.949 |
| mean_IET | income_rec | income_rec | 8.569 | 2 | 0.014 | 0.124 |
| mean_IET | tradeoff_spend_ac | tradeoff_spend_ac | 3.216 | 1 | 0.073 | 0.378 |
| mean_IET | out_work | out_work | 0.202 | 1 | 0.653 | 0.871 |
| mean_IET | HS_comorb | HS_comorb | 1.703 | 1 | 0.192 | 0.668 |
| mean_IET | age | age | 0.557 | 1 | 0.455 | 0.739 |
| mean_IET | AZSVI_Score | AZSVI_Score | 0.064 | 1 | 0.801 | 0.949 |
| max_hourly_IET | res_type | res_type | 0.303 | 2 | 0.859 | 0.949 |
| max_hourly_IET | ac_central | ac_central | 0.243 | 1 | 0.622 | 0.861 |
| max_hourly_IET | lim_ac_use | lim_ac_use | 0.833 | 1 | 0.361 | 0.739 |
| max_hourly_IET | fan_use | fan_use | 3.204 | 1 | 0.073 | 0.378 |
| max_hourly_IET | self_hadap | self_hadap | 1.501 | 2 | 0.472 | 0.739 |
| max_hourly_IET | home_own | home_own | 1.188 | 1 | 0.276 | 0.674 |
| max_hourly_IET | income_rec | income_rec | 16.040 | 2 | 0.000 | 0.012 |
| max_hourly_IET | tradeoff_spend_ac | tradeoff_spend_ac | 6.636 | 1 | 0.010 | 0.120 |
| max_hourly_IET | out_work | out_work | 1.612 | 1 | 0.204 | 0.668 |
| max_hourly_IET | HS_comorb | HS_comorb | 2.381 | 1 | 0.123 | 0.491 |
| max_hourly_IET | age | age | 1.476 | 1 | 0.224 | 0.673 |
| max_hourly_IET | AZSVI_Score | AZSVI_Score | 8.067 | 1 | 0.005 | 0.081 |
| min_hourly_IET | res_type | res_type | 1.572 | 2 | 0.456 | 0.739 |
| min_hourly_IET | ac_central | ac_central | 0.270 | 1 | 0.603 | 0.861 |
| min_hourly_IET | lim_ac_use | lim_ac_use | 1.163 | 1 | 0.281 | 0.674 |
| min_hourly_IET | fan_use | fan_use | 0.012 | 1 | 0.913 | 0.949 |
| min_hourly_IET | self_hadap | self_hadap | 1.507 | 2 | 0.471 | 0.739 |
| min_hourly_IET | home_own | home_own | 0.164 | 1 | 0.686 | 0.881 |
| min_hourly_IET | income_rec | income_rec | 0.104 | 2 | 0.949 | 0.949 |
| min_hourly_IET | tradeoff_spend_ac | tradeoff_spend_ac | 0.023 | 1 | 0.881 | 0.949 |
| min_hourly_IET | out_work | out_work | 0.032 | 1 | 0.857 | 0.949 |
| min_hourly_IET | HS_comorb | HS_comorb | 0.024 | 1 | 0.877 | 0.949 |
| min_hourly_IET | age | age | 0.431 | 1 | 0.511 | 0.767 |
| min_hourly_IET | AZSVI_Score | AZSVI_Score | 2.465 | 1 | 0.116 | 0.491 |

Table S3. Results of Type II omnibus Wald χ² tests evaluating associations between vulnerability factors and daily personal heat exposure (PHE) metrics. Separate mixed-effects models were fitted for each combination of PHE metric and vulnerability factor. Raw p-values were adjusted for multiple testing using the Benjamini–Hochberg false-discovery-rate (FDR) procedure across all omnibus tests (n = 36). Factors with q < 0.05 were considered significant after FDR correction, whereas factors with 0.05 ≤ q < 0.10 were considered suggestive.

Figure S4. Participant-level diurnal cycles of observed 1-h IEH by measurement wave period (left), with regional P_v_ (kPa) shown as dashed black lines. Right: Individual IEH diurnal cycles with markers for participants living in areas with high AZSVI scores (grey squares), self-reported income <$40k/year (black crosses), and participants selected as having high overall exposure (gold), strong diurnal amplitude (red), or stable day-long exposure (violet). Solid lines and shaded bands represent the hourly median ± IQR. None of the participants from wave 1 were retained after data screening.

Figure S5. Participant-level diurnal cycles of observed ∆IET (IET – Regional T_db_) by measurement wave (left), solid black lines indicate IET = Regional T_db_. Markers and color descriptions are as in Figure S4, and lines and shaded bands represent the hourly median ± IQR.

Figure S6. Participant-level diurnal cycles of observed ∆IEH (IEH – Regional P_v_) by measurement wave (left), solid black lines indicate IEH = Regional P_v_. Markers and color descriptions as S4, and lines and shaded bands represent the hourly median ± IQR.

Figure S7. IET deviation (ΔIET: IET − regional T_db_) estimates from the GAM-IET model (See Figure 4c-d). Panel (a) shows individual-level adjusted predictions (CI=95%) centered at the dataset mean regional T_db_ (36.56 °C) and time of day (12h). Panel (b) presents adjusted 24-h predictions from the regional T_db_ diurnal cycle, illustrating ΔIET inter-individual differences as if participants were exposed to the same regional weather. Colors in (c) and (d) indicate selected participants by having the highest overall exposure (gold), the strongest diurnal amplitude (red), or stable day-long exposures (violet), participants not included in any of the groups (black).

Figure S8. Personal heat stress estimates sensitivity to varying wind speed. 2D histograms indicating required evaporati heat losses to achieve heat balance ($\boldsymbol{E}_{\boldsymbol{req}}$) (y-axis) and corresponding maximum potential on evaporative loss at skin ($\boldsymbol{E}_{\boldsymbol{max}_{\boldsymbol{lim}}}$: $\boldsymbol{min}\mathbf{(}\boldsymbol{E}_{\boldsymbol{max}\mathbf{,}\boldsymbol{env}}\mathbf{;}\boldsymbol{E}_{\boldsymbol{max}\mathbf{,}\boldsymbol{sweat}}$)) (x-axis) estimated from 1-h observed PHE and wind speed at (a) 0.3 m/s, (b) 0.8 m/s, (c) 2.3 m/s. 0.3 and 0.8 m/s correspond to low and higher limits for thermal comfort for sedentary occupants at indoors (ASHRAE, 2020). 2.3 m/s corresponds to the 50th percentile of the estimated wind speed at 1.5 m using the power-law profile from KPHX records at 10 m. Estimates of $\boldsymbol{E}_{\boldsymbol{req}}$ were stratified by average awake-sleep self-reported periods with metabolic heat production at M = 1.5 MET (sedentary to light activity) for awake and M=1 MET (rest) for sleep. The histograms are divided into bins of 10 W/m^2^, and the grey lines indicate null heat exchanges. All estimates are conservative, as higher activity intensities or additional radiative load (e.g., sun exposure) would increase $\boldsymbol{E}_{\boldsymbol{req}}$. Median $\boldsymbol{E}_{\boldsymbol{req}}$ fluctuates from 14.5 w/m^2^ (wind at 0.3 m/s), 4.6 w/m^2^ (wind at 0.8 m/s), and to -8.4 w/m^2^ (wind at 2.3 m/s), indicating even less need or no need for evaporative heat losses at a higher wind speed. Median Emax increased with air flow from 121.4,162.7, to 200.1 w/m^2^, still considerably higher than $\boldsymbol{E}_{\boldsymbol{req}}$ corresponding to metabolic heat generated by light activities.

Figure S9. Personal heat stress estimates sensitivity to varying maximal sweat rate. Similar as Figure S8 with estimates of $\boldsymbol{E}_{\boldsymbol{req}}$ and $\boldsymbol{E}_{\boldsymbol{max}_{\boldsymbol{lim}}}$at (a) 0.4 l/h as a low bound value, (b) 0.63 l/h as an average value in Vanos et al. (2023), (c) 0.8 l/h as a high bound value for maximal sweat rate. Minimal changes are due to $\boldsymbol{E}_{\boldsymbol{max}_{\boldsymbol{lim}}}$ being restricted more from $\boldsymbol{E}_{\boldsymbol{max}\mathbf{,}\boldsymbol{env}}$ (environmental restrictions and proportion of body surface with sweat-saturated skin) than $\boldsymbol{E}_{\boldsymbol{max}\mathbf{,}\boldsymbol{sweat}}$(limits on physiological sweat production rate).

Bibliography

ASHRAE. (2020). *ANSI/ASHRAE Standard 55-2020: Thermal Environmental Conditions for Human Occupancy*.

Bailey, E., Fuhrmann, C., Runkle, J., Stevens, S., Brown, M., & Sugg, M. (2020). Wearable sensors for personal temperature exposure assessments: A comparative study. *Environmental Research*, *180*, 108858. https://doi.org/https://doi.org/10.1016/j.envres.2019.108858

Guardaro, M., Hondula, D. M., Ortiz, J., & Redman, C. L. (2022). Adaptive capacity to extreme urban heat: the dynamics of differing narratives. *Climate Risk Management*, *35*(February), 100415. https://doi.org/10.1016/j.crm.2022.100415

Guzman-Echavarria, G., & Caldwell, A. R. (2026). *gguzmane/PHE_HeatSuitePhoenix: V1.0.0 [Software]*. Zenodo. https://doi.org/10.5281/zenodo.19012700

Habeeb, D., Clawson, J., Zakeresfahani, A., & Holtz, Z. (2022). Investigating and Validating On-body Temperature Sensors for Personal Heat Exposure Tracking. *Conference on Human Factors in Computing Systems - Proceedings*. https://doi.org/10.1145/3491102.3517631

Harris, P. A., Taylor, R., Minor, B. L., Elliott, V., Fernandez, M., O’Neal, L., McLeod, L., Delacqua, G., Delacqua, F., Kirby, J., & Duda, S. N. (2019). The REDCap consortium: Building an international community of software platform partners. *Journal of Biomedical Informatics*, *95*, 103208. https://doi.org/https://doi.org/10.1016/j.jbi.2019.103208

Hondula, D. M., Kuras, E. R., Betzel, S., Drake, L., Eneboe, J., Kaml, M., Munoz, M., Sevig, M., Singh, M., Ruddell, B. L., & Harlan, L. (2021). Novel metrics for relating personal heat exposure to social risk factors and outdoor ambient temperature. *Environment International*, *146*, 106271. https://doi.org/10.1016/j.envint.2020.106271

Larsen, L., Gronlund, C. J., Ketenci, K. C., Harlan, S. L., Hondula, D. M., Stone, B., Lanza, K., Mallen, E., Wright, M. K., & O’Neill, M. S. (2022). Safe at Home?: A Comparison of Factors Influencing Indoor Residential Temperatures During Warm Weather Among Three Cities. *Journal of the American Planning Association*, *0*(0), 1–13. https://doi.org/10.1080/01944363.2022.2087724

Vanos, J., Guzman-Echavarria, G., Baldwin, J. W., Bongers, C., Ebi, K. L., & Jay, O. (2023). A physiological approach for assessing human survivability and liveability to heat in a changing climate. *Nature Communications*, *14*(1), 1–14. https://doi.org/10.1038/s41467-023-43121-5

Wright, M. K., Hondula, D. M., Chakalian, P. M., Kurtz, L. C., Watkins, L., Gronlund, C. J., Larsen, L., Mallen, E., & Harlan, S. L. (2020). Social and behavioral determinants of indoor temperatures in air-conditioned homes. *Building and Environment*, *183*(August), 107187. https://doi.org/10.1016/j.buildenv.2020.107187
